# Converging evidence for reduced global atmospheric oxidation in 2020

**DOI:** 10.1093/nsr/nwaf232

**Published:** 2025-06-02

**Authors:** Wei Chen, Yuzhong Zhang, Ruosi Liang

**Affiliations:** College of Environmental and Resource Sciences, Zhejiang University, Hangzhou 310058, China; Key Laboratory of Coastal Environment and Resources of Zhejiang Province, School of Engineering, Westlake University, Hangzhou 310030, China; Institute of Advanced Technology, Westlake Institute for Advanced Study, Hangzhou 310024, China; Key Laboratory of Coastal Environment and Resources of Zhejiang Province, School of Engineering, Westlake University, Hangzhou 310030, China; Institute of Advanced Technology, Westlake Institute for Advanced Study, Hangzhou 310024, China; College of Environmental and Resource Sciences, Zhejiang University, Hangzhou 310058, China; Key Laboratory of Coastal Environment and Resources of Zhejiang Province, School of Engineering, Westlake University, Hangzhou 310030, China; Institute of Advanced Technology, Westlake Institute for Advanced Study, Hangzhou 310024, China

**Keywords:** atmospheric oxidation, hydroxyl radical, methane, COVID-19 lockdowns, wildfire

## Abstract

The hydroxyl radical (OH) plays a central role in the oxidation of methane and many other reduced gases in the Earth's atmosphere. The inter-annual changes of global mean OH concentrations are poorly constrained, posing challenges to understanding annual methane budgets. Here, we use observations of multiple atmospheric species to investigate the anomaly of global OH concentration in 2020. We develop a new method to infer the zonal distribution of OH concentrations from satellite carbon monoxide (CO) observations. This method finds that the global OH concentration reduced by 4.0% ± 0.9% in 2020 relative to 2018–2019 and that the reduction occurred in both Northern (2.4% ± 1.2%) and Southern (5.7% ± 1.2%) Hemispheres. This result is also consistent with our analysis of methane and methyl chloroform observations. Atmospheric chemistry simulations suggest that the OH reduction in the Northern Hemisphere is primarily explained by reduced reactive nitrogen emissions during COVID-19 lockdowns and in the Southern Hemisphere partly by enhanced reactive carbon emissions from extreme Australian fires, hence resulting in distinct chemical mechanisms for OH reduction in the two hemispheres. This contrast is further supported by opposite anomalies in hemispheric tropospheric ozone observed by satellite. Our results highlight the critical role of both anthropogenic and natural perturbations in reduced atmospheric oxidation and rapid methane increases in 2020. This has important implications for future scenarios, given the projected decrease in anthropogenic emissions and increase in fire emissions.

## Teaser

Observations indicate a decline in atmospheric oxidation across both hemispheres in 2020, triggered by COVID-19 lockdowns and the extreme Australian wildfire.

## INTRODUCTION

The hydroxyl radical (OH) is the main oxidant in the troposphere, which controls the removal of reduced gases (e.g. methane, nitrogen oxides, carbon monoxide, volatile organic compounds) and the production of oxidized species (e.g. ozone, strong acids, organic aerosols). Despite its complex dependence on chemical and meteorological conditions, the global mean OH concentration is rather stable, with an inter-annual variability typically less than 5% [1]. However, even such small variability in the global OH concentration can have a large impact on the budgets of a number of key atmospheric species. For instance, a slight reduction in global OH concentration, in the order of a few percent, can result in a considerable perturbation to the global budget of methane, a potent greenhouse gas [2].

Our limited ability to detect and quantify inter-annual changes in the global OH concentration has become a major challenge in interpreting the global methane budget. This is shown in a recent debate on the drivers of the extreme 14.8 ppb a^−1^ methane growth in 2020, as compared to average annual increase of 7.6 ppb a^−1^ in the last decade [3]. Some studies have suggested that the extreme growth was driven by reduced global OH because of the reduction of NO_x_ emissions during the COVID-19 lockdowns [4–7], some attributed it to mainly increases in wetland methane emissions [8–13], while some others found that both factors contributed equally [14]. Additionally, substantial differences in methodologies and analytical frameworks across these studies hinder direct quantitative comparison. Similarly, studies also disagree on whether changes in emissions or OH are responsible for the stabilization of atmospheric methane in the early 2000s [15–17] and for the resumed growth after 2007 [18–24].

Global OH concentrations can be estimated using an atmospheric chemistry model that simulates tropospheric photochemistry [25–28]. However, this approach is subject to the uncertainties of representing the complex OH chemistry and its dependent factors (e.g. anthropogenic and natural emissions of NO_x_, carbon monoxide (CO) and volatile organic compounds (VOCs), as well as meteorological and radiative conditions) in a model, leading to substantial discrepancies in OH concentrations across models [25,27] (Fig. S1) and lower interannual OH variability compared to observation proxies [26,28]. Improvements can be made by the assimilation of chemical observations (e.g. NO_2_, CO and HCHO), often provided by satellite [29–34], and incorporating more advanced chemical mechanisms [35–37].

Alternatively, global mean OH concentration and its inter-annual and decadal changes are often inferred from measurements of ‘proxy’ gases that are removed or produced primarily by OH reactions, e.g. methyl chloroform (MCF) [1,38], ^14^CO [39,40], hydrofluorocarbons (HFCs) and hydrochlorofluorocarbons (HCFCs) [41,42], methane (CH_4_) [43,44] and formaldehyde (HCHO) [45]. MCF is the most widely used proxy. However, these proxy methods often rely on accurate emissions information that is not always available. More importantly, many of these proxies, intended to detect the global mean concentration, are insensitive to the spatial-temporal distribution of the changes [46] because of their long atmospheric lifetime, making it difficult to link detected global OH changes to natural or anthropogenic perturbations that usually occur on regional and seasonal scales [45].

Here, we present an observation-based multi-species analysis to investigate the anomaly of atmospheric oxidation in 2020. This includes a new method based on satellite CO observations that infers tropospheric OH concentrations by season and latitude band, and reduces reliance on detailed chemical mechanisms. Additionally, changes in tropospheric OH concentrations are also analyzed independently using MCF and methane observations. We further interpret the results from these OH proxies through a series of atmospheric model simulations in combination with observed tropospheric ozone anomalies. These analyses provide consistent evidence for reduced global atmospheric oxidation in 2020 and, moreover, shed light on the mechanisms behind the reduction and the impact on rapid 2020 methane growth.

## RESULTS AND DISCUSSION

### Reduced 2020 global OH concentrations inferred from atmospheric observations

**Figure 1.**
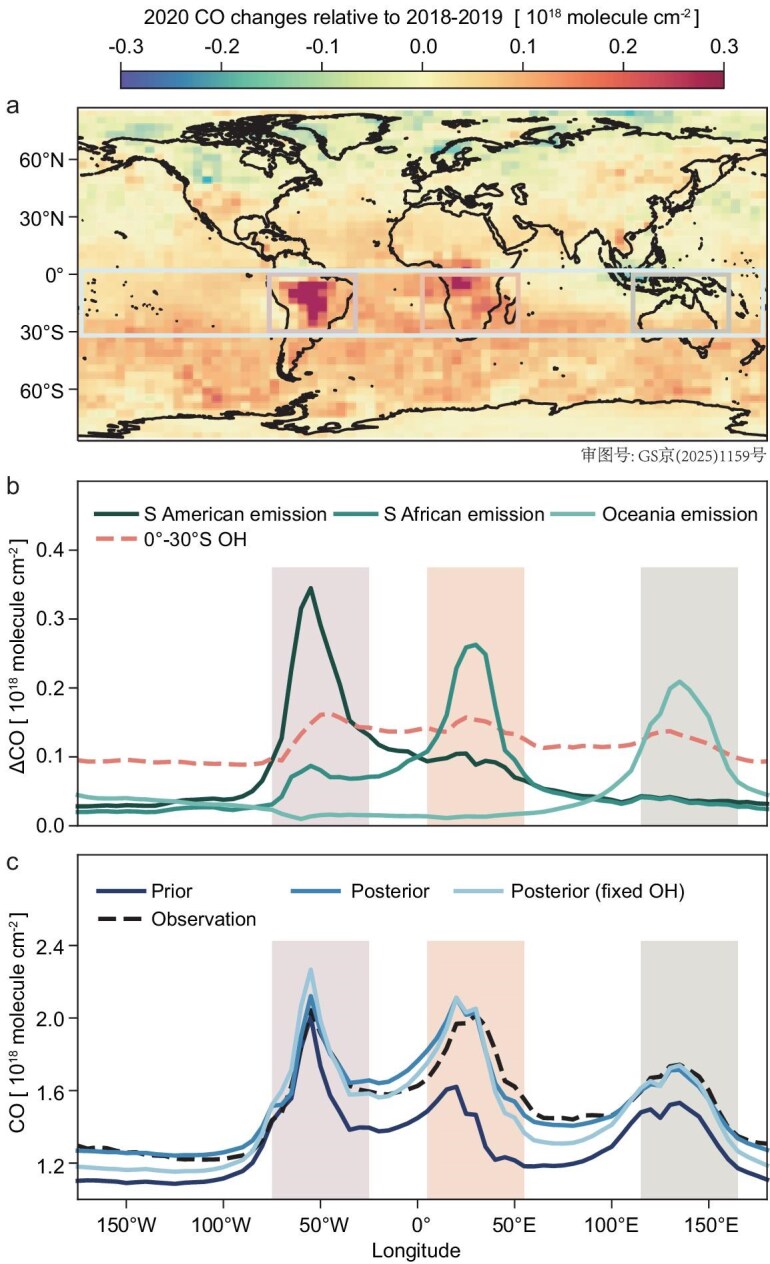
CO column densities and their sensitivities to continental emissions and zonal OH concentration. (a) Changes of CO columns in 2020 relative to 2018–2019 measured by the MOPITT satellite instruments as the difference in annual mean column concentration mapped on a 4° × 5° grid. (b) Sensitivities of CO columns to continental emissions from South America, South Africa and Oceania, and to zonal OH concentration in 0°–30°S. (c) Comparison of the observed CO longitudinal distribution in 0° – 30°S with prior and posterior (with and without optimization of OH concentrations) simulations.

We develop a new method to infer the latitudinal distribution of tropospheric OH concentrations from satellite observations of CO. The method jointly solves seasonal CO emissions from 21 continental regions (Fig. S2) and OH concentrations in six latitude bands, by separating the distinct signatures of CO sources and sinks from the continent–ocean concentration gradients observed by the satellite (Fig. 1) (see Methods and Supplementary Text 1–2).

**Figure 2.**
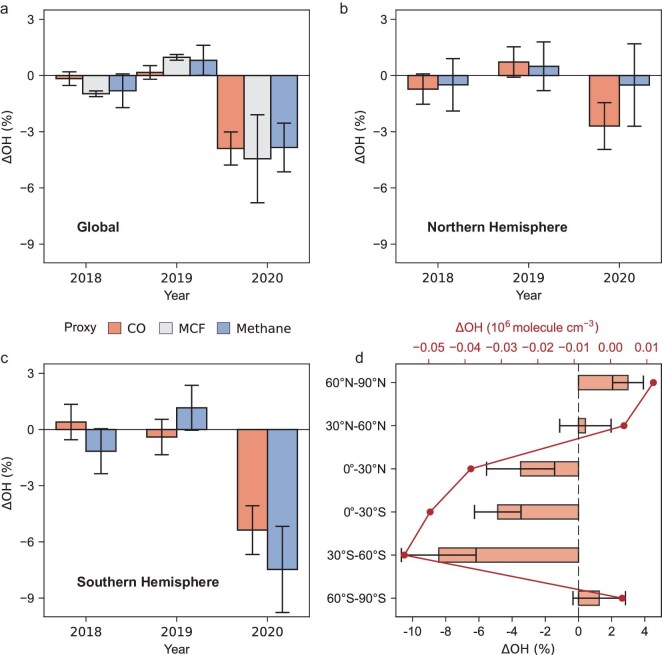
Global and hemispheric OH concentration relative to the 2018–2019 mean inferred from atmospheric observations of CO, MCF and methane. (a) Global. (b) Northern Hemisphere. (c) Southern Hemisphere. (d) Latitudinal OH changes in 2020 relative to 2018–2019 inferred from atmospheric observations of CO.

Figure 2 shows global OH concentrations inferred from the satellite CO method, along with results from other OH proxies. The satellite CO method finds a reduction of global OH concentrations by 4.0% ± 0.9% in 2020 relative to 2018–2019. This reduction is primarily informed by substantially higher CO concentrations over remote oceans (particularly the Southern Ocean) in 2020 than 2018–2019 (Fig. 1a), which cannot be solely explained by enhanced CO emissions given observation constraints over land. This can only be reconciled with a joint adjustment of the zonal OH concentration, which leads to improved agreement with satellite CO observations over both continents and oceans (Fig. 1b and c), and against both observations used in the analysis (MOPITT) and those for independent evaluation (TROPOMI) (Fig. S3). Meanwhile, our method also infers a reduction of CO emissions from major economies in 2020 (see more discussions in Supplementary Text 3), consistent with independent studies on changes in reduced anthropogenic emissions during COVID-19 lockdowns [47,48], supporting the robustness of the CO method.

Our results from CO observations are generally consistent with our analyses of surface MCF observations [1,38,49–51] (see Methods and discussion in Supplementary Text 4) and satellite methane observations (see Methods and discussion in Supplementary Text 5). We infer a reduction of global OH concentration by 4.4% ± 2.1% from MCF data and by 3.8% ± 1.4% from methane data (Fig. 2a). The strong consistency among these independent methods provides compelling observational evidence for the reduction in global OH concentrations in 2020. This finding of reduced 2020 OH is also qualitatively consistent with recent studies based on model simulations and proxy observations (e.g. NO_2_, HFC/HCFC; Supplementary Text 6) [4–7,11,42].

In addition to the global OH estimate, the CO method also provides latitudinal information on OH changes (Fig. 2d), which is usually challenging for conventional proxies such as MCF. This sensitivity to zonal OH concentrations benefits from extensive satellite observation coverage, as well as comparable timescales between the chemical loss of CO (1–3 months) and mixing within a latitude band. Our analysis of CO observations shows reduced 2020 OH concentrations in both the Northern and Southern Hemispheres (Fig. 2b and c). The reduction in the Southern Hemisphere (5.7% ± 1.2%) is greater than that in the Northern Hemisphere (2.4% ± 1.2%). Similar results are also found by our inverse analysis of satellite methane observations (Northern Hemisphere: 0.5% ± 2.4%; Southern Hemisphere: 7.5% ± 2.4%) (Fig. 2b and c; Supplementary Text 5). The significant OH decline in the Southern Hemisphere is also supported by an independent analysis of ^14^CO observations [40]. Previous studies attribute the 2020 OH reduction mainly to decreased anthropogenic NO_x_ emissions during COVID-19 lockdowns [4–7,14]. This implies a predominantly Northern Hemisphere reduction, given that most emission reductions occurred there [4,14,47,48] (Fig. S4). However, our observation-based analysis reveals that OH concentrations decreased across both hemispheres in 2020, with an even more pronounced decline in the Southern Hemisphere, indicating that factors beyond COVID-19 lockdowns played a substantial role.

### Attribution of reduced 2020 OH concentrations

We perform atmospheric chemical transport simulations to explore the drivers for reduced OH concentrations in 2020. In addition to COVID-19 lockdowns, we also consider another notable anomaly, the extreme Australian wildfires [52–55]. The impact of the COVID-19 lockdowns was most pronounced in the Northern Hemisphere (Fig. S4), whereas the Australian wildfires predominantly affected the Southern Hemisphere. The impacts of both events on OH peak during the warm season of their respective hemispheres (Fig. S5). We use the GEOS-Chem model that accounts for the tropospheric O_3_-NO_x_-VOC-HO_x_ chemistry and incorporate findings from recent studies on anthropogenic emissions during the COVID-19 lockdowns [47] and fire emissions from the Australia extreme event [52,56,57] (see Methods for more information on simulation experiments).

**Figure 3.**
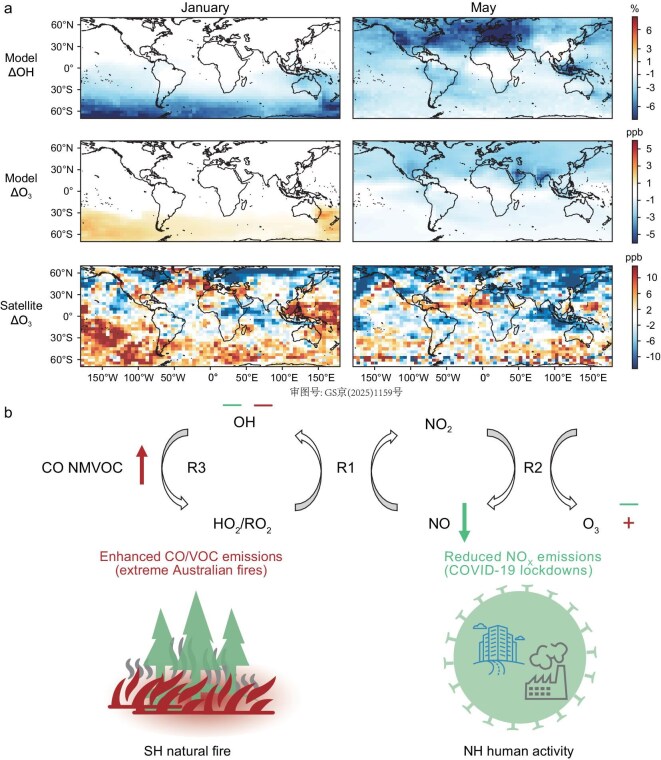
Contrasting OH and O_3_ responses to reduced human emissions and enhanced natural fire emissions. (a) Chemical responses to perturbations by the Australian wildfires in January (left) and COVID-19 lockdowns in May (right). Model responses are computed as the difference between the baseline and sensitivity simulation (see Methods). Observed tropospheric ozone responses are computed by subtracting the monthly average of 2019 from that of 2020. (b) Schematic presentation of a simplified chemical mechanism governing O_3_ and OH responses to emission perturbations by Australian fires and COVID-19 lockdowns. Arrows represent main perturbations to precursor emissions by the two events; + and − signs represent positive and negative chemical responses, respectively. Red symbols are for Australian fire, and green for COVID-19 lockdowns.

Figure 3a shows the effects of COVID-19 lockdowns and the Australian wildfires on simulated OH concentrations. The GEOS-Chem simulations show that COVID-19 lockdowns led to a 2.0% reduction globally in May, with approximately 80% of this reduction occurring in the Northern Hemisphere (Fig. 3a). In contrast, the Australian wildfires led to a 2.0% OH reduction globally in January, predominantly impacting the Southern Hemisphere (Fig. 3a). These results suggest that both COVID-19 lockdowns and the extreme Australian wildfires were substantial contributors to reduced OH concentrations in 2020 inferred from atmospheric observations.

Moreover, the simulation predicts that reduced OH concentration is associated with decreased tropospheric ozone in the Northern Hemisphere during April–July but with increased tropospheric ozone in the Southern Hemisphere during January–March. These ozone anomalies predicted by simulations are consistent with satellite [58,59] and ozonesonde [60] observations. For instance, satellite observations by IASI + GOME2 [58] show a 2020-to-2019 decrease of tropospheric ozone at 3 km by 3.1 ppb in the Northern Hemisphere in April–July and an increase by 2.3 ppb in the Southern Hemisphere in January–March (Fig. 3a and Fig. S6).

These contrasting responses of OH and ozone (O_3_) in the two hemispheres are explained by chemical mechanisms illustrated in Fig. 3b (see more discussions in Supplementary Text 7). Briefly, reduced NO_x_ emissions due to COVID-19 lockdowns led to a slower reaction between NO and HO_2_/RO_2_ (R1), which then resulted in slower OH regeneration (R1) (lower OH concentration) and slower ozone production from NO_2_ photolysis (R2) (lower O_3_ concentration) [4] (Fig. 3b). In contrast, enhanced CO and non-methane VOC (NMVOC) emissions from Australian wildfires rapidly consumed OH but at the same time produced HO_2_ (R3), which then led to a faster production of NO_2_ (R1) and subsequently ozone (R2) (higher O_3_ concentration). Although R2 regenerates a fraction of OH consumed in R3, the net effect is that the photochemical equilibrium within the HO_x_ (≡ OH + HO_2_) family moves away from OH towards HO_2_ (lower OH concentration) [61,62] (Fig. 3b). Consequently, the differing responses in tropospheric ozone between the two hemispheres underscore the distinct chemical mechanisms that govern the reduction of OH concentrations, further supporting our attribution of 2020 OH reductions to both COVID-19 lockdowns and Australian wildfires.

Our model simulations provide insights into the drivers for the 2020 OH decline across both hemispheres. However, these simulations still underestimate the 2020 OH reduction compared to our inference based on atmospheric observations, particularly in the Southern Hemisphere. Based on the simulation, COVID-19 lockdowns and Australian fires together explain 2% OH decrease in the Northern Hemisphere and 2% decrease in the Southern Hemisphere (Fig. S5), compared to 2.4% and 5.7% inferred from the CO method (Fig. 2). This result suggests that additional factors may also have contributed to the observed reduced OH in 2020, for instance, fire aerosol injection into the stratosphere [55,63], increased isoprene emissions [64] and reduced lightning activity [65,66]. However, these factors are not captured by our simulations. Additionally, the simulation is subject to model errors in percussor emissions and chemical mechanisms [25–27,37]. The simulated changes in OH concentrations due to Australian wildfires are sensitive to both the magnitude (fire activity) and the carbon-to-nitrogen ratio of fire emissions (fire combustion conditions) (Fig. S7), both of which are identified as uncertain by recent studies on this fire event [52,56,57]. Similarly, uncertainties persist regarding the overall extent of anthropogenic emission reductions and the relative reduction between reactive carbon and nitrogen species during COVID-19 lockdowns [4,6,7,14]. These challenges in accurately modeling OH concentrations underscore the critical need for top-down constraints on atmospheric oxidation capacity, as demonstrated in this study, through multi-species observations.

### Implications for methane growth in 2020

**Figure 4.**
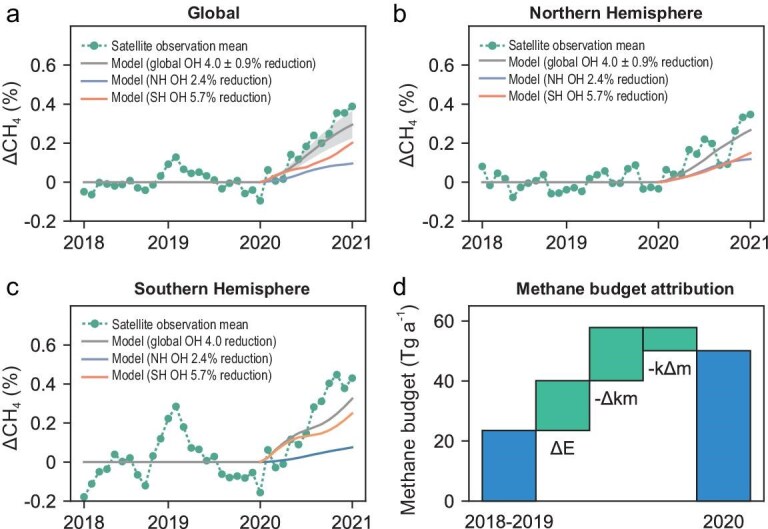
Implications of OH reduction for the methane surge in 2020. Impact of OH reduction on global (a) and hemispheric (b and c) methane growth anomalies in 2020, and anomalies observed from the GOSAT satellite instrument (green lines) are compared with those simulated by the GEOS-Chem CH_4_-only model driven by reduced OH globally (gray), in the Northern Hemisphere (blue), and in the Southern Hemisphere (orange), respectively. Derivation of growth anomalies is illustrated in Fig. S9. (d) The changes in the global methane budget between 2018–2019 and 2020 are decomposed into contributions of changing emissions (Δ*E*), OH (−Δ*km*), and methane burdens (−*k*Δ*m*). Δ*E* and −Δ*km* are considered as forcing terms while −*k*Δ*m* represents relaxation to the steady state. See Supplementary Text 8 for detailed discussion on the decomposition.

The substantial reduction of the global OH concentration, consistently inferred from multiple observations, has significant implications for understanding the drivers of the large methane growth in 2020. Global average atmospheric methane concentrations observed at the National Oceanic & Atmospheric Administration (NOAA) surface network increased by 14.8 ppb in 2020, one of the largest annual growth rates on record [3]. In comparison, the annual growth rate was 8.7 ppb a^−1^ in 2018 and 9.6 ppb a^−1^ in 2019 [3]. Figure 4 compares growth rate anomalies in column methane concentrations measured by the GOSAT instrument [67] to that predicted by a GEOS-Chem CH_4_-only simulation (Table S1) driven by reduced OH inferred by the CO method (with emissions fixed). The comparison shows that substantial OH reduction occurred in both hemispheres, as inferred by the CO method (Fig. 4b and c), can explain a large fraction of enhanced methane growth rates, which is also observed in both hemispheres [5,68]. In contrast, the enhanced methane growth rates in both hemispheres cannot be as well captured if reduced OH occurred mainly in the Northern Hemisphere driven by COVID-19 lockdowns [4–7,14] (Fig. S8).

We further quantify the contribution of reduced OH concentration to the extreme growth observed in 2020 based on methane inversion results, which provide a consistent breakdown of the global methane budget (see Supplementary Text 8 for discussion of the methodology and comparison with the literature). We find an excessive methane increase of 27 Tg a^−1^ in 2020 relative to 2018–2019, which is contributed to by both enhanced methane emissions (17 Tg a^−1^) and reduced OH concentrations (18 Tg a^−1^), offset by enhanced chemical removal in response to increased methane concentrations (−8 Tg a^−1^) (Fig. 4d). The increase in methane emissions is mainly due to tropical and high-latitude wetlands [8–10,14] (Fig. S10). This analysis shows that the reduction of OH in 2020 accounts for 52% ± 19% (54% ± 12% if based on CO proxy) of the forcing on the 2020 methane budget compared to the 2018–2019 budget (see Supplementary Text 8). Previous studies have shown discrepancies in the 2020 methane growth rates derived from surface observations and satellite inversions [8,9,14]. Our analysis of methane inversion suggests that this discrepancy can be resolved by accounting for the variation in the conversion ratio between the total atmospheric burden and surface concentrations (Fig. S11 and Fig. S12; see Supplementary Text 8). Applying a constant average conversion ratio to observed surface mixing ratio changes, as done in some previous studies, may have led to an underestimation of the methane burden growth anomaly in 2020. The use of a constant conversion factor implicitly assumes that methane is well mixed in the troposphere, which is sufficiently good for multi-year or longer timescales (Fig. S12a) but is not accurate enough for inter-annual variations considered here (Fig. S12b). Given the projection of decreases in anthropogenic emissions and increases in fire emissions [69–72], our study implies a growing role of chemistry–climate interactions in regulating the global methane budget through atmospheric oxidation capacity in the future.

## METHODS

### Inference of tropospheric OH concentrations from observations

We develop a method to infer tropospheric OH concentrations by season and latitude band from satellite CO observations (Fig. S13). We briefly describe the justification and the procedure below, with more information provided in Supplementary Text 1. We perform an observing system simulation experiment (OSSE) to evaluate the ability of this method to capture the changes in seasonal OH concentrations due to varied drivers. The setup and the results of the OSSE are described in Supplementary Text 2.

We consider that the atmospheric concentration of CO (${n}_{\rm CO}$) is governed by the following equation:


(1) \begin{eqnarray*}
\frac{{d{n}_{\rm CO}}}{{dt}} &=& - \nabla \cdot ({n}_{\rm CO}{\boldsymbol{u}}) + \tilde{E} - \tilde{L} + {\mathrm{minor\ terms\ }}\\
&\approx & - \nabla {\boldsymbol u}\cdot{n}_{\rm CO} + E + \mathop \sum \limits_i {\gamma }_i{E}_i\\
&&- \left( {{k}_{\rm CO}{n}_{\rm CO} - {k}_{\rm C{{\mathrm{H}}}_4}{n}_{\rm C{\rm H}_4}} \right){n}_{\rm OH},
\end{eqnarray*}

which consists of (i) a tracer transport term $ - \nabla \cdot ({n}_{\rm CO}{\boldsymbol{u}})$ (${\boldsymbol{u}}$ is the wind vector); (ii) an OH-independent term ($\tilde{E}$) representing primary emissions (*E*) and secondary production from short-lived VOCs ($\mathop \sum \nolimits_i {\gamma }_i{E}_i$); and (iii) an OH-dependent term ($\tilde{L}$) representing removal by oxidation against tropospheric OH, which are partially offset by secondary production from long-lived VOCs (mainly methane). The term $\tilde{L}$ can be expressed as $( {{k}_{\rm CO}{n}_{\rm CO} - {k}_{\rm C{{\mathrm{H}}}_4}{n}_{\rm C{\rm H}_4}} ){n}_{\rm OH}$ and is proportional to the OH concentration (${n}_{\rm OH}$) (${k}_{\rm CO}$ is the rate constant of CO + OH, ${k}_{\rm C{{\mathrm{H}}}_4}$ is the rate constant of CH_4 _+ OH, and ${n}_{\rm C{\rm H}_4}$ is the concentration of methane). Within the spatial scale of interest here, the contribution of short-lived VOC *i* to $\tilde{E}$ can be effectively modeled as the product of its CO yield (${\gamma }_i$) and emissions (${E}_i$) and hence is independent of ${n}_{\rm OH}$ (see Table S2 for ${\gamma }_i$ values), because they are rapidly oxidized upon emission. This assumption is examined in Fig. S14 (the shape of blue and green lines), which shows that short-lived VOC oxidation treated as instantaneous emissions leads to CO signatures that closely match those predicted by the full-chemistry simulation.

We note that $\tilde{E}$ and $\tilde{L}$ in Eq. (1) exert distinct signatures on the distribution of CO concentrations along a latitude band (Fig. 1b and Fig. S14), because $\tilde{E}$ occurs predominantly over the continents, while by comparison $\tilde{L}$ is more uniform throughout the troposphere along a latitude band. This contrast in their signatures suggests that the observed continent–ocean gradient of CO may be exploited to separate the signal of latitude-band-averaged OH concentrations from that of continental-scale CO emissions. This also provides insights into previous findings that atmospheric CO observations are useful to inform regional and global OH concentrations [29,73].

Built on the above analysis, we develop an inversion algorithm (Supplementary Text 1), which assimilates CO column densities measured by the MOPITT instrument on board the NASA/Terra satellite (Level 3 product v8) [74] (see ‘CO observations’ in Supplementary Text 1 for more information). The dataset demonstrates good quality over both land and water/ocean regions, with no significant systematic biases between these environments [75,76]. This is crucial for the algorithm, as the concentration gradient between continental and remote ocean areas contains essential information that enables the separation of emissions and OH concentrations within a latitude band (see Supplementary Text 1). We optimize $\tilde{E}$ from 21 terrestrial regions (Fig. S2) and average OH concentration (${n}_{\rm OH}$) in six latitude bands (i.e. 90°S–60°S, 60°S–30°S, 30°S–0°, 0°–30°N, 30°N–60°N and 60°N–90°N). The inversion is solved analytically and recursively every 3 months for 2018–2020. The forward model for the inversion is provided by the ‘CO-only’ simulation of the GEOS-Chem chemical transport model (v12.9.3) (Table S1). This simulation treats the OH field as an exogenous parameter (decoupling of CO and OH chemistry) and solves Eq. (1) on a 4° × 5° global grid. The simulation is driven by the MERRA-2 reanalysis meteorological field [77] to realistically capture the transport.

The prior estimate for $\tilde{E}$ is given by bottom-up emission inventories including the community earth atmospheric data system (CEDS) for anthropogenic emissions [78], the global fire emissions database (GFED4s) for fire emissions [57] and the model of emissions of gases and aerosols from nature (MEGAN) for biogenic VOC emissions [79]. The optimization of $\tilde{E}$ by the inversion corrects prior biases in both primary CO emissions and secondary formation from reactive VOCs (including biases in specified yield values), though they are not separable by the algorithm. To characterize the uncertainty, we perturb emission inventories and yield values in sensitivity inversions. We also assess the error arising from the approximation that reactive VOCs are instantaneously oxidized (Fig. S15a).

The prior estimate for OH is based on 3D monthly OH fields from full-chemistry model simulations. We solve for season-averaged OH concentrations in six latitude bands and do not seek to optimize the OH distribution on smaller spatial and temporal scales (e.g. horizontal and vertical distributions within a latitude band). Error in the prior OH distribution on finer scales can therefore lead to biases in the posterior estimates [43]. To examine this potential error, we construct an ensemble of inversions using 14 prior OH fields (with monthly 3D distributions) generated from chemical transport models with different setups and chemical mechanisms (11 from the ACCMIP project and an additional three from GEOS-Chem v5, GEOS-Chem v11 and GEOS-Chem v12) [25,80] (Fig. S15a). These OH fields are intended to capture a reasonable range of uncertainties in the horizontal and vertical distributions of prior OH concentrations (Fig. S1).

We report the inferred changes in global and hemispheric OH concentrations based on the ensemble mean of a series of sensitivity inversions, where model inputs and inversion assumptions are perturbed (Fig. S15a), to mitigate any systematic errors in the method. To quantify the uncertainty of the reported values, we calculate the standard deviation of the sensitivity inversions. Briefly, there are five groups of sensitivity inversions, including (i) prior OH distributions; (ii) prior anthropogenic and fire emissions; (iii) the CO yield from isoprene oxidation; (iv) the spatial distribution of secondary CO production; and (v) error statistic specifications. Our analysis identifies prior OH distributions as the most critical factor influencing the uncertainty of OH inference, followed by the error statistic specifications (Fig. S15a).

In addition, we analyze the posterior error covariance matrix (Eq. S3 in Supplementary Text 1) to understand the error structure of the solution. Figure S16 shows the error correlation derived from ${{\bf \hat{S}}}$ (posterior error covariance matrix; see Supplementary Text 1). There are some moderate positive error correlations between regional emissions and latitude-band OH and weak negative error correlations between OH in adjacent latitude bands. Overall, this result suggests that the method can reasonably separate the signals from emissions and OH. Furthermore, aggregating by hemisphere, the error correlations between the estimates of the Northern and Southern Hemisphere OH concentrations range from −0.08 to −0.02, smaller than those of the methane method (between −0.11 and −0.2), indicating better separation of hemispheric OH by the CO method.

We also use *in situ* measurements of MCF, an ozone-depleting compound banned by the Montreal Protocol, from a global network of 14 surface sites to infer the inter-annual variability of tropospheric OH concentrations in 2018–2020. The MCF method is a conventional observation-based approach to derive global OH levels [1,38,81,82]. See Supplementary Text 4 for a detailed description of the MCF method.

In addition, we perform an inverse analysis of satellite methane observations (GOSAT) [67], incorporating retrievals over both land and ocean (glint mode) in the inversion. This approach estimates hemispheric OH concentrations together with methane emissions on a 4° × 5° grid. The inversion is set up following Maasakkers *et al.* [83] and Zhang *et al.* [84]. See Supplementary Text 5 for a detailed description of the methane method.

### Simulation of OH and ozone responses to 2020 COVID-19 lockdowns and Australian wildfires

We use global atmospheric chemistry simulations to examine the response of global OH concentration and its distribution to suppressed anthropogenic emissions during COVID-19 lockdowns and to the extreme fire event in Australia. Our simulations are performed on a 4° × 5° grid with the ‘tropchem’ simulation by the GEOS-Chem chemical transport model (v12.9.3) (Table S1) driven by the MERRA-2 re-analyzed meteorology [77]. The ‘tropchem’ mechanism describes tropospheric chemistry with ∼200 species (including O_3_, HO_x_, NO_x_, CO, VOCs and aerosol compositions) and ∼800 reactions (Table S1). This ‘full-chemistry’ simulation allows us to explicitly simulate OH concentrations and their feedback to the chemical environment, unlike in the ‘CO-only’ and ‘CH_4_-only’ simulation discussed above, where a prescribed OH field is used to intentionally decouple OH from CO or CH_4_. Hence, the ‘full-chemistry’ simulation serves as a tool for examining the mechanisms underlying the findings of our top-down analyses.

In this study, the baseline simulation is driven by business-as-usual anthropogenic emissions compiled from regional inventories for Asia (MIX-Asia v1.1) [85], the USA (2011 National Emissions Inventory, NEI-2011), Canada (Air Pollutant Emission Inventory, APEI) and Africa (DICE-Africa) [86] and a global inventory (CEDS) [78] for the rest of the world. For aviation emissions, we use Aviation Emissions Inventory Code (AEIC) [87,88]. For natural emissions, we use GFED4s for wildfires [57], MEGAN for biogenic VOCs [79], and the parameterization of Murray *et al.* [89] for lightning NO_x_. Soil NO_x_ emissions are from Hudman *et al.* [90].

In a sensitivity simulation, we apply anthropogenic emissions derived from a dataset by Lamboll *et al.* [47], which accounts for the impact of COVID-19 lockdowns on anthropogenic emissions, including those from aircraft. Lamboll *et al.* [47] report decreases of 15.3%, 12.5% and 9.7% relative to the business-as-usual scenario for anthropogenic NO_x_, CO and short-lived VOC emissions, respectively. Their results also indicate that roughly 86.5% of the decrease occurred in the Northern Hemisphere and that the largest decrease occurred in April–June 2020. We compute the response of OH to COVID-19 lockdowns as the difference between this sensitivity simulation and the baseline simulation.

In another sensitivity simulation, we exclude the extreme fire emissions in Australia spanning November 2019–January 2020 to isolate their impact. The total emissions from the extreme Australian fire event amounted to 76.7 Tg CO, 0.6 Tg NO and 3.5 Tg C short-lived VOC, which is an order of magnitude higher than fire emissions in the region over the past 16 years [52,56,57]. We compute the response of OH to Australian fires as the difference between the baseline simulation and the sensitivity simulation without emissions from this fire event. The effect of the uncertainty in fire emissions (emission magnitude and the carbon-to-nitrogen emission ratio) on the simulated OH response is shown in Fig. S7.

To better understand the chemical nature of OH variations, we also examine tropospheric ozone, another key species of atmospheric chemistry, with our simulations. We compare the simulated responses of tropospheric ozone to the above perturbations with anomalies observed by satellite observations of tropospheric ozone at 3 km, retrieved jointly from GOME-2 measurements in the ultraviolet band and IASI measurements in the infrared band [58]. The IASI + GOME-2 system is shown to have high retrieval sensitivity to ozone in the lower troposphere (e.g. 3 km) over both land and ocean [58]. This analysis of tropospheric ozone offers further insights into the contrasting chemical mechanisms driving OH reductions in both hemispheres.

### Analysis of 2018–2020 global methane budgets with observation-constrained OH concentrations

We demonstrate the impact of reduced OH on the global and hemispheric methane concentrations by applying the OH concentrations inferred from the CO method to a GEOS-Chem CH_4_-only simulation during 2018–2020. The simulation results are processed and then compared to GOSAT satellite observations [67] following the method described as Laughner *et al.* [5] to illustrate the contribution of reduced OH concentration in 2020 to observed global and hemispheric methane growth.

We also use the methane inversion described above to quantify the respective contributions of reduced OH concentrations and increased methane emissions to the rapid increase of atmospheric methane in 2020, which allows for a consistent comparison with results reported in the literature, despite significant methodological differences (see Supplementary Text 8 for discussion on methane budget breakdown and the comparison with literature).

## Supplementary Material

nwaf232_Supplemental_File

## Data Availability

The MOPITT v8 TIR-NIR CO product is available at https://asdc.larc.nasa.gov/data/MOPITT/MOP03J.008. NOAA surface MCF and methane observations are accessible through NOAA ESRL/GMD (https://gml.noaa.gov). The IASI + GOME2 ozone satellite data are from https://iasi.aeris-data.fr/o3_iago2. The GOSAT CO_2_ proxy methane observations are available at https://catalogue.ceda.ac.uk/uuid/18ef8247f52a4cb6a14013f8235cc1eb. The 2020 anthropogenic emissions applied in the COVID-19 lockdowns sensitivity simulation are from https://zenodo.org/record/4736578#.Y9dYEXZByUk. Code and documentation for the GEOS-Chem model is available at http://geos-chem.seas.harvard.edu.
