# Supplementary material for: Converging evidence for reduced global atmospheric oxidation in 2020
Source: Natl Sci Rev. 2025 Jun 2;12(8):nwaf232. doi: 10.1093/nsr/nwaf232 (PMC12361742; doi:10.1093/nsr/nwaf232)
Supplement: nwaf232_Supplemental_File [file nwaf232_supplemental_file.docx]

**Supplementary Information for “Converging evidence for reduced global atmospheric oxidation in 2020”**

Wei Chen^1,2,3^, Yuzhong Zhang^2,3*^, Ruosi Liang^1,2,3^

^1^College of Environmental and Resource Sciences, Zhejiang University, Hangzhou 310030, China

^2^Key Laboratory of Coastal Environment and Resources of Zhejiang Province, School of Engineering, Westlake University, Hangzhou 310030, China

^3^Institute of Advanced Technology, Westlake Institute for Advanced Study, Hangzhou 310030, China

Correspondence to: Yuzhong Zhang (zhangyuzhong@westlake.edu.cn)

**Contents of this file:**

Supplementary Texts 1 to 8

Supplementary Figures 1 to 19

Supplementary Tables 1 to 5

# Text 1 Inference of OH concentration from inverse analysis of satellite CO observations

## State vector

We design an inverse analysis to infer seasonal latitude-band averaged OH concentrations from satellite CO observations. The inversion seeks to optimize the state vector (the set of variables to be optimized) on a seasonal basis. The state vector includes (1) general CO emissions (including primary emissions and secondary production from short-lived VOCs) from 21 subcontinental regions (21 elements) (Fig. S2) and (2) average tropospheric OH concentration in every 30^o^ latitude band (6 elements).

With this design of the state vector, we attempt to separate and extract the signal of OH (removal of CO by OH offset by production of CO from CH_4_+OH) and general emissions (primary emissions and secondary production from short-lived VOCs) from global coverage of CO observations. The rationale for this design is that a substantial OH perturbation with global significance would likely manifest across a broader region (e.g., a latitude band). For example, full-chemistry simulations show that events like the COVID-19 lockdowns and Australian wildfires can lead to OH perturbations that span entire latitude bands (Fig. 3a). In such cases, the signature of OH perturbation and general emissions on CO concentrations are distinct and thus separable. Figure 1b demonstrates that the response of CO concentrations to perturbed average OH concentrations (the OH signature) is relatively uniform along the 0-30^o^S latitude band. In contrast, the response to perturbed general emissions (the emission signature) is much larger over continents where emission sources are, fading rapidly towards the ocean. Additionally, the relatively uniform response of CO to OH can be further demonstrated in a full-chemistry simulation where NO_x_ emissions are reduced according to the COVID-19 lockdowns scenario, while CO and VOC emissions remain unchanged (in this case, the change in CO concentrations is fully driven by changes in OH) (Fig. S17). Although this results in substantial variations in OH changes across the Northern mid-latitudes (because of spatial variations in NO_x_ emission reduction) (Fig. S17), its impact on the gradient of CO concentrations is still minor (Fig. S17).

The inversion retrieves OH concentrations by each 30^o^ latitude band and by season. This design aims to resolve the strong latitudinal gradient of OH from the tropics to the poles. Previously proposed methods such as the MCF method are designed to resolve global or at best hemispheric OH, because the observations of these proxies are very sparse while these species have a long lifetime (several years) and thus are well mixed in the troposphere, smearing any signals for resolving finer OH distributions. In comparison, CO has a lifetime of 1-3 months, which is comparable to the time scale of atmospheric mixing in a 30^o^ band. This hints that the observed distribution of CO may contain information that can resolve OH concentrations by latitude band. In addition, we choose a 3-month optimization window, a result of a trade-off between capturing the OH variations with time and reducing the effect of the initial conditions.

## CO observations

We use CO column densities retrieved from thermal infrared and near-infrared measurements by the MOPITT instrument onboard NASA/Terra [1] (Level 3 product v8; daily means on a 1^o^×1^o^ global grid). Measurements are made globally over both continents and oceans in a 612 km swath with a 22 km×22 km footprint and a 3-day return time [2]. Morning over-pass observations (around 10:30 local time) are used for analysis.

MOPITT CO retrievals have been extensively evaluated against ground-based remote sensing (e.g., TCCON, NOAA, NDACC) and in situ aircraft measurements (e.g., HIPPO, ATOM, KORUS-AQ) [3-6]. These evaluations show that MOPITT CO retrievals exhibit similar performance over both land and water/ocean regions, with no significant systematic biases between these environments [4, 5], hence robustly capturing the continent-ocean gradient of CO concentrations. Furthermore, the evaluation also shows that the long-term bias drift in MOPITT column data is negligible [1], enabling their application for inter-annual and long-term studies [2, 7].

Additionally, we also use CO retrievals from the TROPOMI instrument to independently evaluate the performance of the inversion (Fig. S3).

## Prior estimates

Prior estimates for total CO emissions are derived from a series of bottom-up inventories. We use CEDS for anthropogenic emissions of CO and reactive VOCs [8], which are then overridden by regional inventories in the US (2011 National Emissions Inventory, NEI-2011), Canada (Air Pollutant Emission Inventory, APEI, 2017), Asia (MIX-Asia v1.1) [9], and Africa (DICE-Africa) [10]. We use the Global Fire Emissions Database (GFED4s) [11] for wildfire emissions. We use biogenic reactive VOC emissions (including isoprene, monoterpenes, methanol, and acetone) from the Model of Emissions of Gases and Aerosols from Nature (MEGAN) [12]. CO production from oxidation of reactive VOCs is treated as instantaneous emissions and computed as the product of precursor emissions and corresponding CO yields [13-15] (Table S2 and Table S3).

Prior estimates for OH concentrations are derived from archived monthly 3-dimensional OH fields from full-chemistry simulations. To capture the uncertainty arising from the OH distributions (in terms of both horizontal and vertical distributions), we perform sensitivity inversions using different OH fields in the forward simulation. 11 of these OH fields are from the ACCMIP project [16] and 3 from various versions of “tropchem” GEOS-Chem simulations [17] (Fig. S1).

## Forward model

We use the “CO only” simulation (Table S1) by the GEOS-Chem chemical transport model version 12.9.3 as the forward model for the inversion, to relate CO concentrations to CO emissions or OH concentrations. The simulation is driven by MERRA-2 reanalyzed meteorology [18] and solves Eq. (1) on a global 4°×5° grid with 47 vertical layers (30 layers in the troposphere). The “CO only” simulation computes CO removal by oxidation against OH and CO production from the CH_4_+OH reaction based on a prescribed OH field. This decoupling of OH from CO and CH_4_ in the simulation is desired, as we want to perturb independently OH concentrations and CO emissions to solve the inversion. In addition, the simulation computes CO production from methane with zonal average tropospheric methane mixing ratios derived from NOAA surface observations [19, 20], to account for the contribution of increasing methane to CO chemical production. The simulation treats secondary production from short-lived VOCs as instantaneous emissions (Table S2 and Table S3). A pretest shows that this approximation incurs only small errors in the CO spatial distribution on the global 4°×5° grid (Fig. S14). To compare with satellite observations, we compute simulated CO columns by applying prior profiles and averaging kernels reported in the satellite data to simulated vertical profiles.

## Inversion procedure

To find the solution to the state vector (***x***), we minimize the following Bayesian cost function [21]:

$$\begin{aligned} J\left( \boldsymbol{x} \right)=\left( \boldsymbol{x}-\boldsymbol{x}_{\boldsymbol{a}} \right)^{T}{\mathbf{S}_{\mathbf{a}}}^{-1}\left( \boldsymbol{x}-\boldsymbol{x}_{\boldsymbol{a}} \right)+{\gamma\left( \boldsymbol{y}-\boldsymbol{F}\left( \boldsymbol{x} \right) \right)}^{T}{\mathbf{S}_{\mathbf{o}}}^{-1}\left( \boldsymbol{y}-\boldsymbol{F}\left( \boldsymbol{x} \right) \right),\#\left( S1 \right) \end{aligned}$$

where $\boldsymbol{x}_{a}$ is the prior estimate of ***x***, ***y*** is the observation vector, and ***F*** is the forward model given by GEOS-Chem. $\mathbf{S}_{a}$ is the prior error covariance matrix and $\mathbf{S}_{o}$ is the observation error covariance matrix. We take $\mathbf{S}_{a}$to be diagonal and assume 50% error standard deviation for continental-scale CO emissions from the 21 clusters and 5% error standard deviation for OH concentrations in the 6 latitude bands. We take $\mathbf{S}_{O}$ to be diagonal and compute the variance terms from statistics of the residual error ($\varepsilon_{O}=y-F\left( x_{a} \right)-\bar{y-F(x_{a})}$, and we find that observation error standard deviations average 2.5×10^17^ molecule cm^-2^ (roughly 20% of the average column). $\gamma$ is the regularization parameter introduced to prevent overfitting from the diagonal assumption of $\mathbf{S}_{o}$ and $\mathbf{S}_{a}$. We determine $\gamma$=0.05 based on the L-curve plot [22]. We linearize the forward model at the prior estimate and construct the Jacobian matrix $\mathbf{K=}\frac{d\boldsymbol{F}(\boldsymbol{x}_{\boldsymbol{a}})}{d\boldsymbol{x}}$ by perturbing each element of the state vector in “CO-only” GEOS-Chem simulations.

The optimal solution ($\hat{\boldsymbol{x}}$) to the linearized Bayesian problem and the corresponding error covariance matrix ($\hat{\mathbf{S}}$) are given by

$$\begin{aligned} \hat{\boldsymbol{x}}=\boldsymbol{x}_{\boldsymbol{a}}+\left( {\gamma\mathbf{K}}^{T}{\mathbf{S}_{\mathbf{o}}}^{-1}\mathbf{K}+{\mathbf{S}_{\mathbf{a}}}^{-1} \right)^{-1}{\gamma\mathbf{K}}^{T}{\mathbf{S}_{\mathbf{O}}}^{-1}\left( \boldsymbol{y}-\boldsymbol{F}\left( \boldsymbol{x}_{\boldsymbol{a}} \right) \right), \#\left( S2 \right) \end{aligned}$$

$$\begin{aligned} \hat{\mathbf{S}}\mathbf{=}\left( \gamma\mathbf{K}^{T}{\mathbf{S}_{\mathbf{o}}}^{-1}\mathbf{K+}{\mathbf{S}_{\mathbf{a}}}^{-1} \right)^{-1}\boldsymbol{. \#}\left( S3 \right) \end{aligned}$$

Because of the $k_{CO}n_{CO}n_{OH}$ term in Eq. (1), this inverse problem is nonlinear. We argue, however, that the non-linearity is only moderate because the prior estimate is close to the true value, and hence Eq. (S2) and (S3) are sufficient for an improved estimate of ***x***.

We solve this inverse system sequentially every 3 months during 2018-2020, taking advantage of the CO lifetime to fully exploit the information embedded in CO observations. The posterior simulation for a 3-month inversion is used to generate the initial condition for the next 3 months. For the initial condition of January 2018, we include a 3-month spin-up inversion from October to December 2017. As shown in Fig. S18, the initial conditions are generally unbiased against satellite observations, and more importantly, consistent with posterior estimates (with no discontinuity in the global CO burden).

# Text 2 Observing system simulation experiment

We design an observing system simulation experiment (OSSE) to evaluate the efficacy of the proposed method, focusing on whether the method can retrieve the changes in the OH concentration driven by various types of perturbations. The OSSE approach allows us to examine the ability of our method to separately constrain $\tilde{E}$ and $n_{OH}$ based on satellite CO observations and a simplified forward model.

Table S4 summarizes the OSSE conditions for the “true” and inversion simulations. We use the “tropchem” mode (Table S1) of the GEOS-Chem chemical transport model to generate the “true” global 3-D distribution of CO and OH concentrations. The simulation explicitly accounts for the complex tropospheric NO_x_-VOC-O_3_-HO_x_-aerosol chemistry described by ~200 species and ~800 reactions and is driven by anthropogenic and natural emissions of these species. The “true” CO concentration field is sampled following the MOPITT product to generate synthetic satellite observations of monthly averaged CO column densities, which are used as input data to the inversion. The “true” OH concentration is then used to evaluate the estimates from the inversion.

Except for observation data from the “true” atmosphere simulation, the inversion follows the method described in Methods and Supplementary Text 1. The “true” atmosphere and inversion simulations differ in several key aspects to reduce the “fraternal twin” problem (Table S4). First, the inversion uses the “CO-only” mode (Table S1) of the GEOS-Chem model as the forward model, which sufficiently describes the budget of atmospheric CO but depends minimally on the mechanism of complex tropospheric chemistry, in contrast to the full-chemistry, “tropchem” used to generate the “true” atmosphere. Second, the inversion simulation is driven by prior estimates of $\tilde{E}$ (anthropogenic and natural CO and VOC emissions) and $n_{OH}$ that are deliberately different from the “true” atmosphere. Finally, we use different meteorological data for the “true” and inversion simulations.

To test the sensitivity of our method to changes in OH concentrations, we generate a series of “true” atmospheres by perturbing the input to “tropchem” simulations relative to a baseline simulation. Table S5 summarizes the setting of these simulations representing varied anthropogenic and natural drivers for the changes in OH concentrations, including anthropogenic emissions (ANTH_NOX_0.75 and ANTH_INC), biogenic isoprene emissions (BIOGENIC_0.9), fire emissions (FIRE_1.25), and lightning NO_x_ emissions (LIGHTNING_0.85). In ANTH_NOX_0.75 and LIGHTNING_0.85, only NO_x_ emissions are perturbed. In BIOGENIC_0.9, only emissions of a reactive VOC isoprene are perturbed. In ANTH_INC and FIRE_1.25, emissions of all emitted species, including NO_x_, CO, and VOC, are perturbed.

Our evaluation focuses on the detection of OH changes at the latitude-band or hemispheric scales. Figure S19 compares inferred ΔOH from synthetic observations with “true” ΔOH from perturbed and baseline simulations. Most of the “true” ΔOHs being tested are between -0.5×10^5^ to 0.5×10^5^ molecules cm^-3^, which are on the order of several percent of the global mean OH concentration (roughly 10×10^5^ molecules cm^-3^). In general, our method can capture these small changes in OH concentrations, regardless of the drivers, with no apparent biases. The root-mean-square error (RMSE) is 0.18×10^5^ molecules cm^-3^ and R^2^ is 0.49 for latitude bands and 0.15×10^5^ molecules cm^-3^ and 0.63 for hemispheres. Performance is slightly improved when results are aggregated for hemispheres, indicating that the errors of the solution are moderately correlated at the latitude band scale.

We conclude from the OSSE that the proposed method can detect and quantify small changes in OH concentrations under various scenarios at the latitude band and the hemispheric scales. However, we note that the OH responses to fire perturbations tend to be overestimated, while those to anthropogenic emission perturbation underestimated (Fig. S19). More investigations are required to understand whether this represents a perturbation-type dependent bias or is due to specific OSSE setup. Moreover, a consistent evaluation of the CO proxy and other OH proxies, as done in this OSSE, would be beneficial for identifying the strengths and limitations of each method, which represents a key direction for future research.

# Text 3 Inference of changes in anthropogenic CO emissions

In addition to OH concentrations, the CO methods also provide optimized estimates for CO emissions. These results are valuable for understanding changes in anthropogenic emissions in 2020 and serve as a validation check for our inversion system and the resulting OH estimates.

The CO method infers reduced annual CO anthropogenic emissions in 2020 from India, China, and Europe by 4.2 ± 0.9%, 2.4 ± 1.3%, and 7.3 ± 2.6%, respectively, relative to the 2018-2019 average. This result is generally expected because of reduced anthropogenic activities during the COVID-19 pandemic. Based on bottom-up calculations, Lamboll et al. [23] and Doumbia et al. [24] also reported reduced CO emissions over these regions linked to COVID-19 lockdowns, with an even greater magnitude (~10%).

The agreement of inferred CO emissions with independent studies lends support to our CO inversions, and subsequently, our inferences of OH variations.

# Text 4 2020 OH reduction inferred from methyl chloroform measurements

We derive 2018-2020 variations in global OH concentrations with in situ flask measurements of methyl chloroform (MCF) measurements from the NOAA surface network [25, 26].

The tropospheric average OH concentration ($n_{OH}$, mole cm^-3^) can be derived from changes in global mean MCF concentrations ($n_{\mathrm{MCF}}$, mole cm^-3^), based on the following equation:

$$\begin{aligned} n_{OH}=\frac{1}{k_{\mathrm{MCF}}{\cdot n}_{\mathrm{MCF}}}\left( \frac{E}{f}-\frac{dn_{\mathrm{MCF}}}{dt} \right)\#\left( S4 \right) \end{aligned}$$

where $E$ is the emission rate of MCF (g s^-1^), $k_{\mathrm{MCF}}$ is the rate constant of the MCF+OH reaction ((mole cm^-3^ s)^-1^), $f$ is a constant that converts emitted mass to tropospheric concentrations (g (mole cm^-3^)^-1^), and $\frac{dn_{\mathrm{MCF}}}{dt}$ represents the time tendency of global mean MCF concentrations. We assume that $k_{\mathrm{MCF}}$ is constant inter-annually, so relative changes in $n_{OH}$ are independent of the value of $k_{\mathrm{MCF}}$.

We first compute monthly global average MCF concentrations ($n_{\mathrm{MCF}}$) based on monthly average concentrations measured at 14 background sites across the globe. We test three different averaging methods. (1) We compute latitude-band averages by grouping 14 sites into 6 30^o^-latitude bands and take the average from these 6 values (Band-avg); (2) We perform the same calculation as (1) except that a weighted average is taken for the global mean with a weight of 0.4, 0.76, and 0.97 for high, middle, and low latitudes, respectively following Montzka et al. [26] (Band-Wtavg); (3) We group sites by hemisphere instead of by latitude band before calculating global averages (Hemi-avg). We take a 1-year window moving average to remove seasonal cycles in the monthly global average time series and then take a central difference to compute monthly time tendency of global MCF concentrations.

In addition to different averaging methods, we also test 7 varied scenarios of MCF emissions to assess their impact on the results. Four scenarios are taken from Patra et al. [27], extrapolated from 2015-2019 to 2020, and the other three derived from the mean and standard deviation reported in Liang & Rigby et al. [28]. All these sensitivity analyses agree on the reduction of global OH concentration in 2020, with the magnitude of the reduction more sensitive to specified MCF emissions than the averaging methods (Fig. S15b). The magnitude of MCF emissions ($E$) is small, as MCF is a banned compound by the Montreal Protocol. However, as the atmospheric concentration of MCF decreases, the inference of global OH concentration becomes increasingly more sensitive to remaining emissions [27]. The uncertainty of the MCF method is reported as the standard deviation of these sensitivity calculations.

Our analysis of MCF shows a 4.4 ± 2.1% reduction in global OH concentrations in 2020 relative to 2018-2019 means (Fig. 2a), generally consistent with our findings from the satellite CO analysis. Figure S15b shows that derived OH reduction is insensitive to varied methods to compute global averages but highly sensitive to the magnitude and the trend of MCF emissions. The strong dependence of the MCF analysis on the small yet uncertain MCF emissions also underscores the value of alternative methods such as the CO method developed in this study.

# Text 5 2020 OH reduction inferred from satellite methane observations

We also perform an inverse analysis of satellite methane observations to estimate hemispheric OH concentrations together with methane emissions on a 4°×5° grid for 2018*–*2020. A recent assessment by Penn et al. [29] demonstrates that satellite methane observations are useful for constraining hemispheric OH concentrations.

Briefly, we use the University of Leicester version 9 retrieval of column methane mixing ratios from the satellite GOSAT instrument [30]. Both retrievals over both land and ocean (glint mode) are used in the inversion. The forward model for the inversion is the GEOS-Chem CH_4_-only simulation, which treats the OH field as an exogenous parameter (Table S1). The simulation is driven by MERRA-2 reanalysis meteorological fields [18]. The prior estimates of methane emissions and OH concentrations and their error statistics are specified following Zhang et al. [31]. Additionally, we specify an error correlation of 0.5 between the Northern and Southern Hemisphere following Penn et al. [29]. The Bayesian optimization problem is solved analytically similar to the CO inversion, following the method established in Maasakkers et al. [32].

The GEOS-Chem simulation, driven by posterior methane sources and sinks of the inversion, reproduces the inter-annual and seasonal variation of both GOSAT satellite observations (used in the inversion) and NOAA surface observations (used for independent evaluation) (Fig. S11), confirming the successful execution of the inversion.

Our methane inversion finds that the reduction of tropospheric OH concentrations in 2020 relative to 2018-2019 were 3.8 ± 1.4% globally, 0.5 ± 2.4% in the Northern Hemisphere, and 7.5 ± 2.4% in the Southern Hemisphere, which agrees reasonably well with our results from the CO inversion and the MCF analysis (Fig. 2). The uncertainty of OH estimates is assessed by Zhang et al. [33] through an observing system simulation experiment. They identified the prior OH spatiotemporal distribution as a main factor, leading to about 2% variability in the posterior estimates of global OH concentrations. Additionally, here we assess the effects of various inversion configurations on the posterior estimates, including the inversion time window (i.e., annual and seasonal), prior OH concentration errors (i.e., 5%, 10%), and the error correlation between hemispheric OH concentration (i.e., 0, 0.5) (Fig. S15c). We determine the estimate for uncertainty by reporting the larger value between the standard deviation derived from the posterior error covariance matrix and from the inversion ensemble.

In addition to OH, the methane inversion also infers increased methane emissions in 2020 relative to 2018-2019 . The increase amounts to 17 Tg a^-1^ (Fig. 4d, and Fig. S10). A substantial portion of this increase is attributed to wetland regions in Africa, North America, northern Europe, and Siberia (Fig. S10). This finding is consistent with previous top-down inversions [34-36] and bottom-up models that account for dynamic wetland responses to climate variables such as temperature and precipitation [35, 37].

The methane inversion provides a consistent breakdown of factors contributing to the 2020 surge of atmospheric methane. Using the posterior simulation, we compare the methane budget in 2020 with the 2018-2019 average (or 2019), methane emissions increased by 17 (13) Tg a^-1^, methane sinks reduced by 18 (22) Tg a^-1^ due to decreases in OH concentrations but increased by 8 (5) Tg a^-1^ due to increases in methane concentrations in 2020, which results in an enhanced methane imbalance of 27 (30) Tg a^-1^. Independent evidence from CO and MCF observations consistently shows strong OH reduction (in particular in the Southern Hemisphere), enhancing our confidence in the attribution of the 2020 methane surge given by the methane inversion.

# Text 6 Discussion on inferring global OH from satellite NO_2_ observations

Satellite NO_2_ retrievals are of high quality and provide high-resolution information on surface NO_x_ emissions and local-scale OH variability. However, several limitations make satellite NO_2_ less suitable for inferring large-scale (from latitude-band to global) OH changes within the framework of our inversion (single species observation with reduced dependence of the methodology on detailed chemical mechanisms).

NO_x_ is not typically used as a proxy for large-scale OH variability because of its short atmospheric lifetime (usually on the order of hours to days) and hence its concentration gradients are only sensitive to OH variabilities at local scales (e.g., city scale) [38-41]. In contrast, the tracers used in our study (CO, CH_4_, and MCF) have longer chemical lifetimes ranging from months to years, which makes them suitable for inferring zonal-mean or global OH on broad spatial and temporal scales. Additionally, the relationship between NO_2_ and OH also depends on the emissions and concentrations of other species (e.g., VOCs) as well as complex chemical mechanisms between them. Accurately relating NO_2_ to OH therefore requires detailed representation of these species and their chemistry, which introduces additional uncertainties. This is in contrast with the CO method that relies primarily on chemical kinetics of a few reactions (see our analysis in Supplementary Text 1).

Although we do not directly use satellite NO_2_ as a proxy for inferring global OH changes in our analysis, our full-chemistry simulation for COVID-19 lockdowns accounts for the decreased NO_x_ emissions. This simulation shows decreased NO_x_ concentrations over major economies in 2020, which are consistent with satellite NO_x_ observations [42, 43] and multi-species data assimilation [44]. For instance, Miyazaki et al. [44] assimilated satellite NO_2_ observations and other species, with a multi-species data assimilation framework, to constrain NO_x_ emissions and used the inferred changes of NO_x_ emissions in a full-chemistry model to simulate OH. Their results also showed a decline in OH concentrations in 2020, consistent with the OH changes inferred in our study, providing an independent line of observational evidence.

In future work, it may be possible to leverage the strengths of satellite NO_2_ for resolving OH variability at finer spatial scales. This information could help refine the spatial structure of the prior OH field at smaller scales, which could then be integrated with the CO/CH_4_ based approach used in this study to improve the accuracy of global OH estimates due to the uncertainty on prior OH distributions.

# Text 7 Chemical mechanisms of reduced OH concentration in 2020

We interpret the chemical response to perturbations by COVID-19 lockdowns and Australian fires with a framework depicted in Figure 3b. This framework employs a highly simplified chemical mechanism compared to what is implemented in GEOS-Chem “tropchem” simulations but is useful to understand the chemistry in the remote troposphere (not applicable for near-source chemistry).

Tropospheric ozone and OH are coupled through the following reaction

$$\begin{aligned} NO+\mathrm{HO}_{2}\to\mathrm{NO}_{2}+OH. \#\left( R1 \right) \end{aligned}$$

R1 is followed by NO_2_ photolysis leading to a net production of tropospheric ozone [45]

$$\begin{aligned} NO_{2}+hv\underset{\to}{O_{2}}NO+O_{3}. \#\left( R2 \right) \end{aligned}$$

Meanwhile, R1 regenerates OH from HO_2_ that are produced from the oxidation of CO, CH_4_, and non-methane VOCs (NMVOC). The regeneration mechanism by R1 constitutes about ~30% of gross OH production in the troposphere [45] and is thus critical for sustaining the atmospheric abundance of OH. The oxidation of CO, CH_4_, and NMVOC by OH often involves complex reaction chains, with the simplest example being CO oxidation:

$$\begin{aligned} CO+OH\underset{\to}{O_{2}}CO_{2}+HO_{2}. \#\left( R3 \right) \end{aligned}$$

CH_4_ and NMVOC oxidation produces RO_2_ which can also react with NO (analogous to HO_2_+NO in R1) to produce NO_2_, leading to net ozone production. Though RO_2_+NO reactions typically do not immediately regenerate OH, further follow-up reactions often produce HO_2_ that contribute to R1.

The relative importance of NO_x_ versus CO and NMVOC emitted from combustion sources are generally determined by combustion efficiency. Anthropogenic emissions, often associated with high-efficiency combustion, tend to have more NO_x_, relative to wildfires that emit more CO and NMVOC. Hence, the COVID-19 perturbation to anthropogenic emissions features a greater reduction in NO_x_ emissions compared to CO and VOCs emissions [23, 24], while the Australian forest fire event is associated with larger enhancements in CO and NMVOC emissions relative to NO_x_ emissions [11, 46, 47]. Both cases result in reduced OH concentrations (Australian wildfires mainly lead to decrease in the Southern Hemisphere while COVID-19 lockdowns decrease in the Northern Hemisphere; Fig. S5) but lead to different O_3_ responses (Fig. 3b).

# Text 8 Discussion on the methodology of attributing changes in the methane growth rate

Differences in methodological details on calculating the fraction of the contribution by emissions or OH to observed methane increases lead to confusion when comparing results between existing studies [48]_._ Here, we discuss two primary methodological issues.

1. **Conversion between surface concentrations and atmospheric burdens.**

Two approaches have been employed to estimate the annual growth of global methane burden for the 2020 case. One is based on in situ surface network and the other is based on the inverse analysis of satellite observations.

**Surface network.** Based on the NOAA surface network, the growth of global average methane concentrations in 2020 is greater than 2019 by 5.2 ppb a^-1^ (growth rate is 14.8 ppb a^-1^ in 2020 and 9.6 ppb a^-1^ in 2019), which is the most widely cited number for the 2020 methane surge. This additional increase of 5.2 ppb a^-1^ in surface concentration is converted to an additional increase of ~15 Tg a^-1^ in the atmospheric methane burden, by applying a constant conversion factor (2.75 Tg ppb^-1^) [49]. Peng et al. [35] made the contribution attribution based on values derived from this approach.

**Satellite inversion.** Atmospheric inversion of satellite methane observations, which directly report global total changes in sources and sinks (in Tg a^-1^), tend to estimate a greater additional increase of the atmospheric methane burden than estimates based on surface network. This study, along with Qu et al. [34], and Feng et al. [36] all inferred a ~30 Tg a^-1^ greater methane increase in 2020 than that in 2019, compared to ~15 Tg a^-1^ derived from the surface network.

It is tempting to attribute this discrepancy between the surface network and the satellite inversion to the different types of observations used. We examine this hypothesis using a simulation driven by methane sources and sinks optimized by our methane inversion. We note that the simulation can reproduce the growth rates of both GOSAT observations and NOAA surface observations (Fig. S11). The difference of annual growth rates between 2020 and 2019 given by this posterior simulation is within 0.5 ppb a^-1^ of that derived from GOSAT or NOAA observations, implicating that results from satellite inversion (30 Tg a^-1^) and NOAA (5.2 ppb a^-1^) are not inconsistent.

Instead, our results suggest the discrepancy results mainly from the use of the constant conversion factor between surface concentrations and burdens. Our posterior simulation of the methane inversion, which captures growth rates of both satellite and surface observations, shows substantial variations (2~3.5 Tg ppb^-1^) in the ratios (i.e., the conversion factor) between annual changes in surface concentrations (sampled at NOAA sites in the model) and annual changes in atmospheric burden (integrated throughout the entire atmosphere) (Fig. S12). The use of a constant conversion factor of 2.75 Tg ppb^-1^ implicitly assumes that methane is sufficiently well mixed in the troposphere. This assumption is sufficiently good when the discussion pertains to multi-year or longer timescales (Fig. S12a) but, as shown in our simulation, is not accurate enough for inter-annual variations considered here (Fig. S12b).

Our posterior simulation of the methane inversion suggests that this ratio (or conversion factor) is lower in 2019 (2.4) but higher in 2020 (3.2) (Fig. S12c), which reconciles the discrepancy in the excessive methane growth derived from surface network and satellite inversion. We thus consider 30 Tg a^-1^ a robust estimation of the changes in global methane budget in 2020 compared to 2018-2019.

1. **Accounting the contributions of emissions and OH to methane growth**

The growth rate of the global methane burden (*G*, in Tg a^-1^) can be described as

$$G=E-km-L,$$

where *E* denotes the global methane emissions (Tg a^-1^), *k* is the loss frequency against oxidation by tropospheric OH (a^-1^), *m* is the total atmospheric methane mass (Tg), and *L* represents minor sinks. If *E* changes by Δ*E*, *k* changes by Δ*k*, and $m$ changes by Δ*m* (assuming changes in *L* are negligible) in the next year, the change in the growth rate ($\Delta G$) between the two years can be expressed as [31] :

$$\Delta G=\left( E+\Delta E \right)-\left[ \left( k+\Delta k \right)\left( m+\Delta m \right)-km \right]=\Delta E-\left( \Delta km+k\Delta m+\Delta k\Delta m \right)\approx\Delta E-\Delta km-k\Delta m,$$

where $\Delta E$ represents the forcing due to the change in emissions and $\Delta km$ represents the forcing due to changes in OH concentration. $k\Delta m$ represents the response of the OH sink to changes in the global methane mass, and therefore $k\Delta m$ is not a forcing term. $\Delta k\Delta m$ is a minor term that can be neglected.

Different ways have been employed in studies to quantify the fractional contributions of OH and emissions:

1. $-(\Delta km+k\Delta m)/\Delta G$
2. $-\Delta km$/$\Delta G$
3. $-\Delta km/(\Delta E-\Delta km)=-\Delta km/(\Delta G+ k\Delta m)$

Although studies often do not explicitly state which method is used, we can deduct from the description that Peng et al. [35] used Method (1), Laughner et al. [50], Stevenson et al. [51] and Skeie et al. [52] used Method (2), and Qu et al. [34] used Method (3).

Method (1) includes the non-forcing *k*Δ*m* term, which is not attributable to OH changes and may therefore underestimate the fractional contribution of OH changes. Method (2) lacks closure of the forcing terms, meaning that the contributions from changes in OH and emissions do not necessarily sum to 1. Method (3) proposed by Qu et al. [34] makes the attribution between the two forcing terms. We use Method (3) in our study. Based on methane inversion results (see Supplementary Text 5), we find that the reduction of OH in 2020 is responsible for 52% (63%) of the 2020 surge, relative to 2018-2019 (2019).


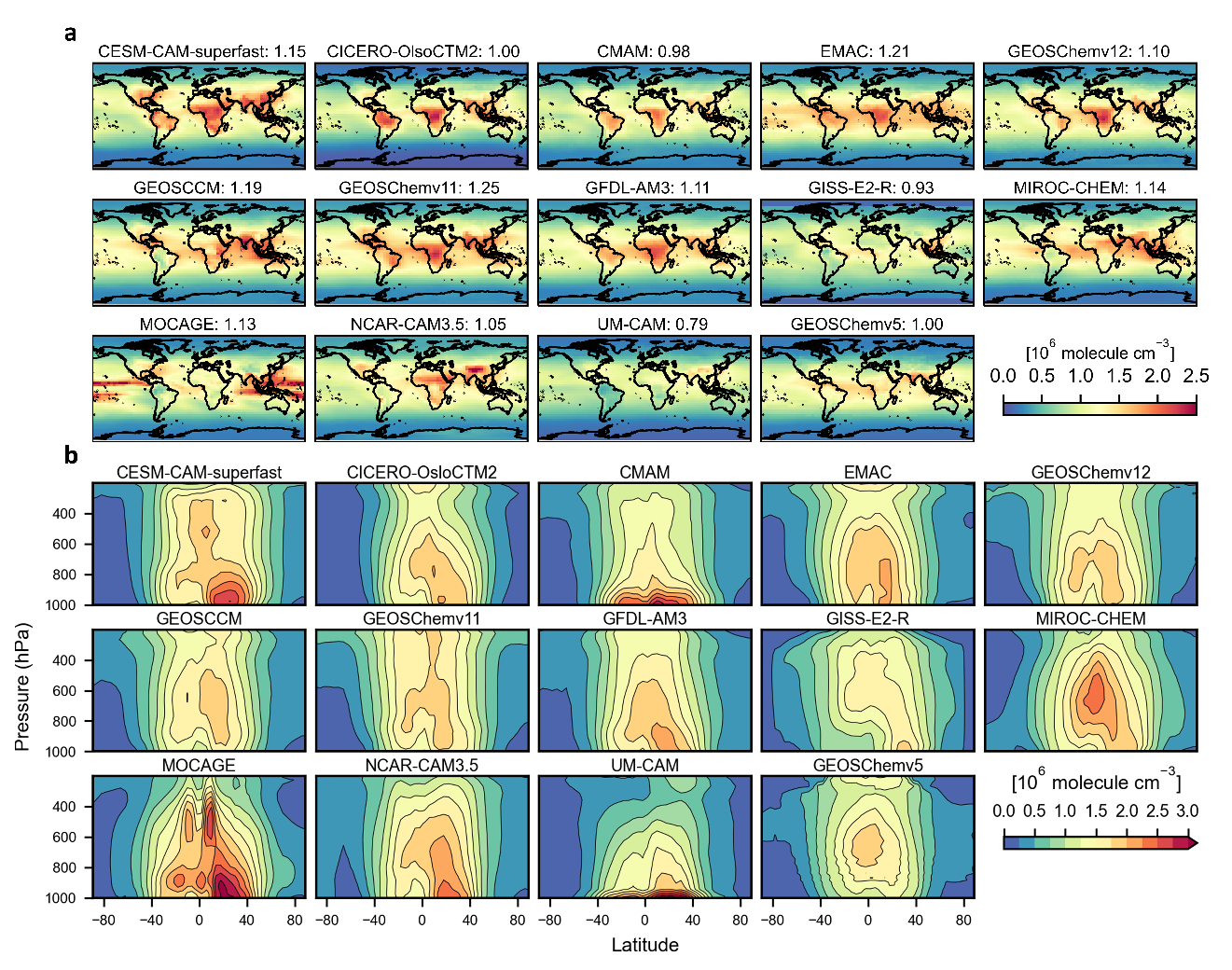


**Figure S1.** OH fields from 14 different global models that are used in the inversion ensemble [16, 17]. a, Horizontal distribution. Annual global mean OH concentrations are shown. b, Vertical distribution.


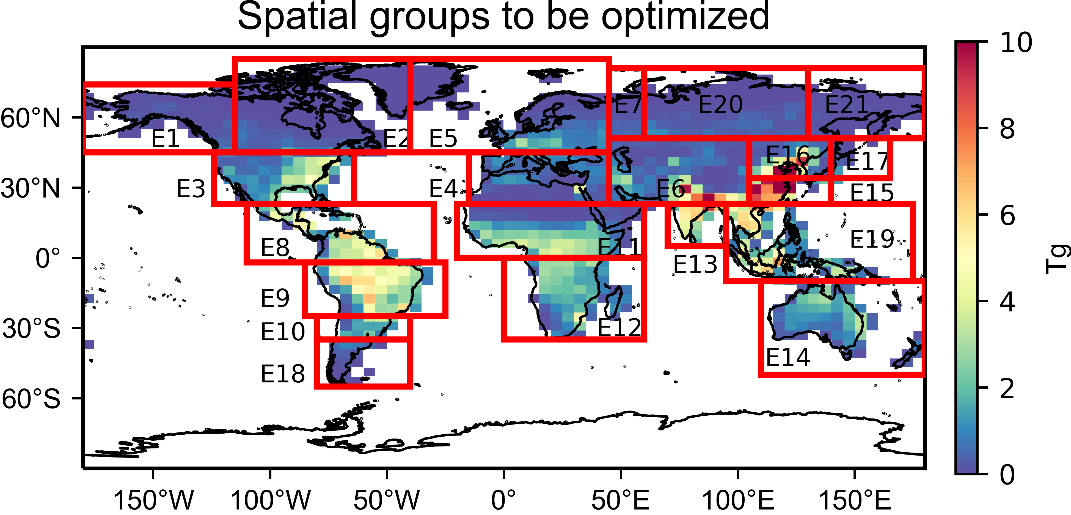


**Figure S2.** Spatial distribution of mean 2018-2020 continent CO source used as prior estimates in the inversion of MOPITT data. Red boxes indicate the 21 subcontinental regions for which total sources are optimized for every 3 months.


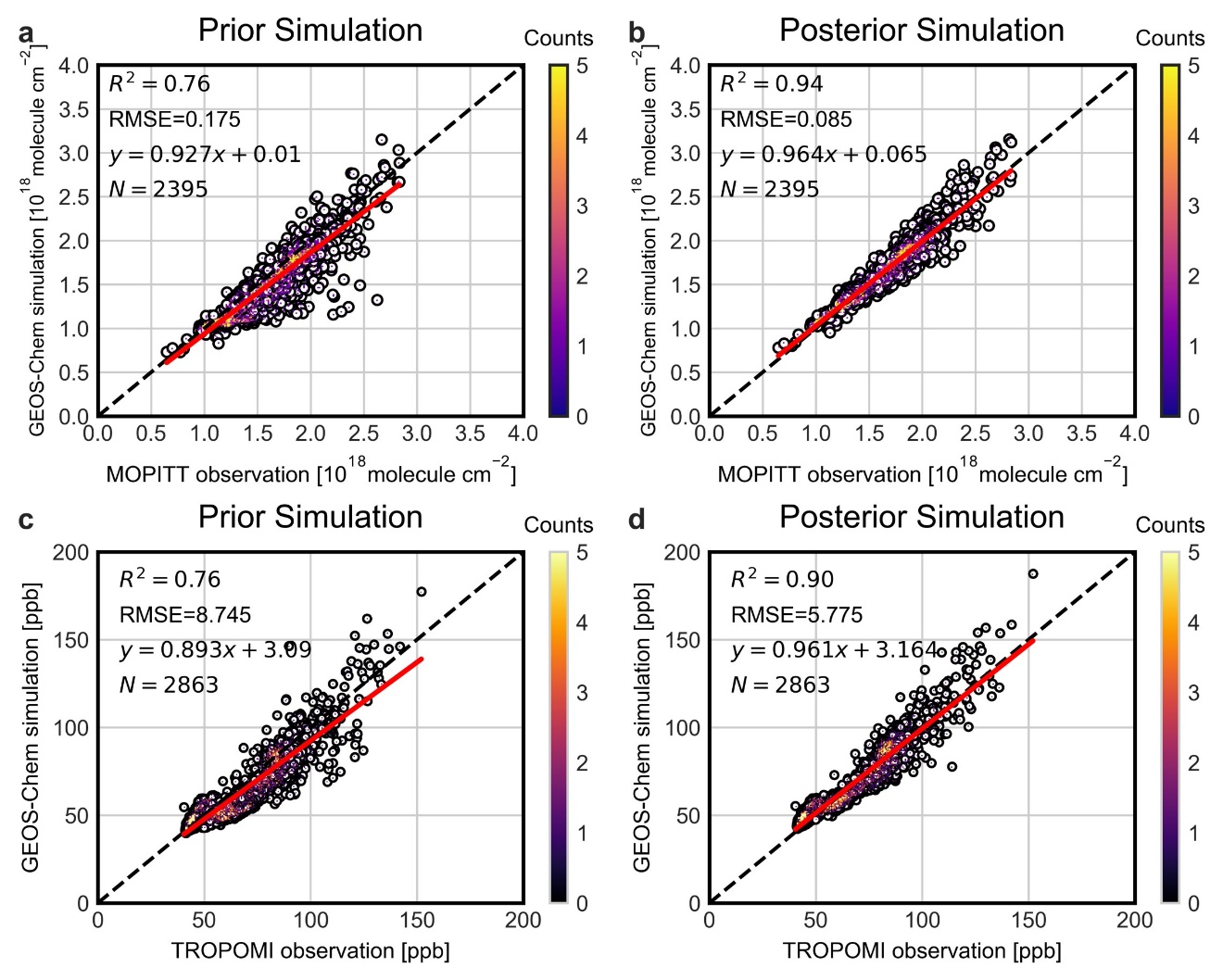


**Figure S3.** Improvement of the fitness to the MOPITT CO observations used in the inverse analysis (a, b) and TROPOMI CO observations for independent evaluation (c, d) averaged on the 4°×5° grid between prior (a, c) and posterior (b, d) simulations.


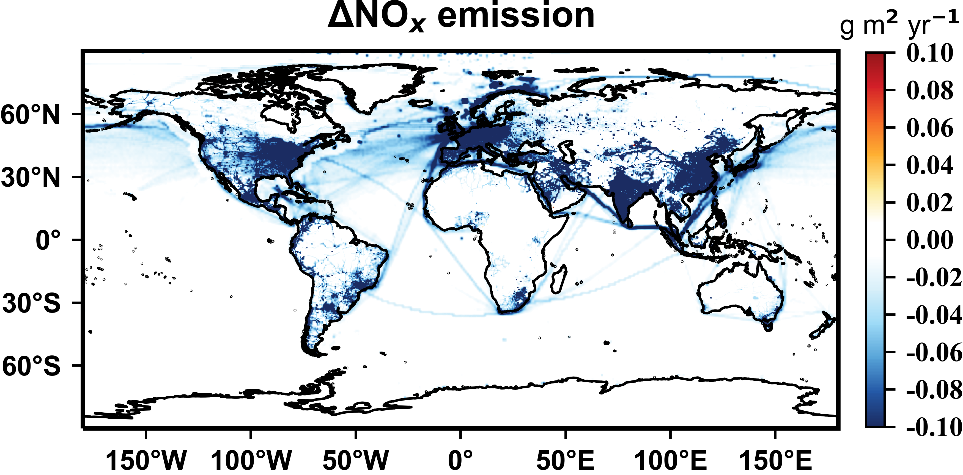


**Figure S4.** Difference in anthropogenic NO_x_ emissions between the baseline and COVID-19 scenarios during 2020 based on Lamboll et al. [23]**.**


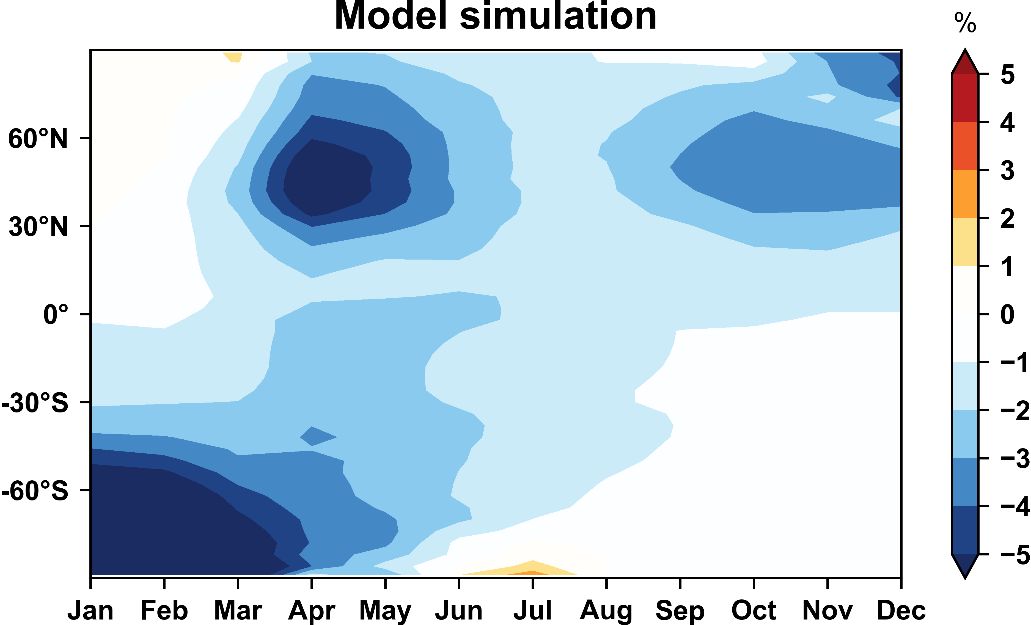


**Figure S5.** Simulated OH anomalies due to COVID-19 lockdowns and Australian wildfires as a function of latitude and month.


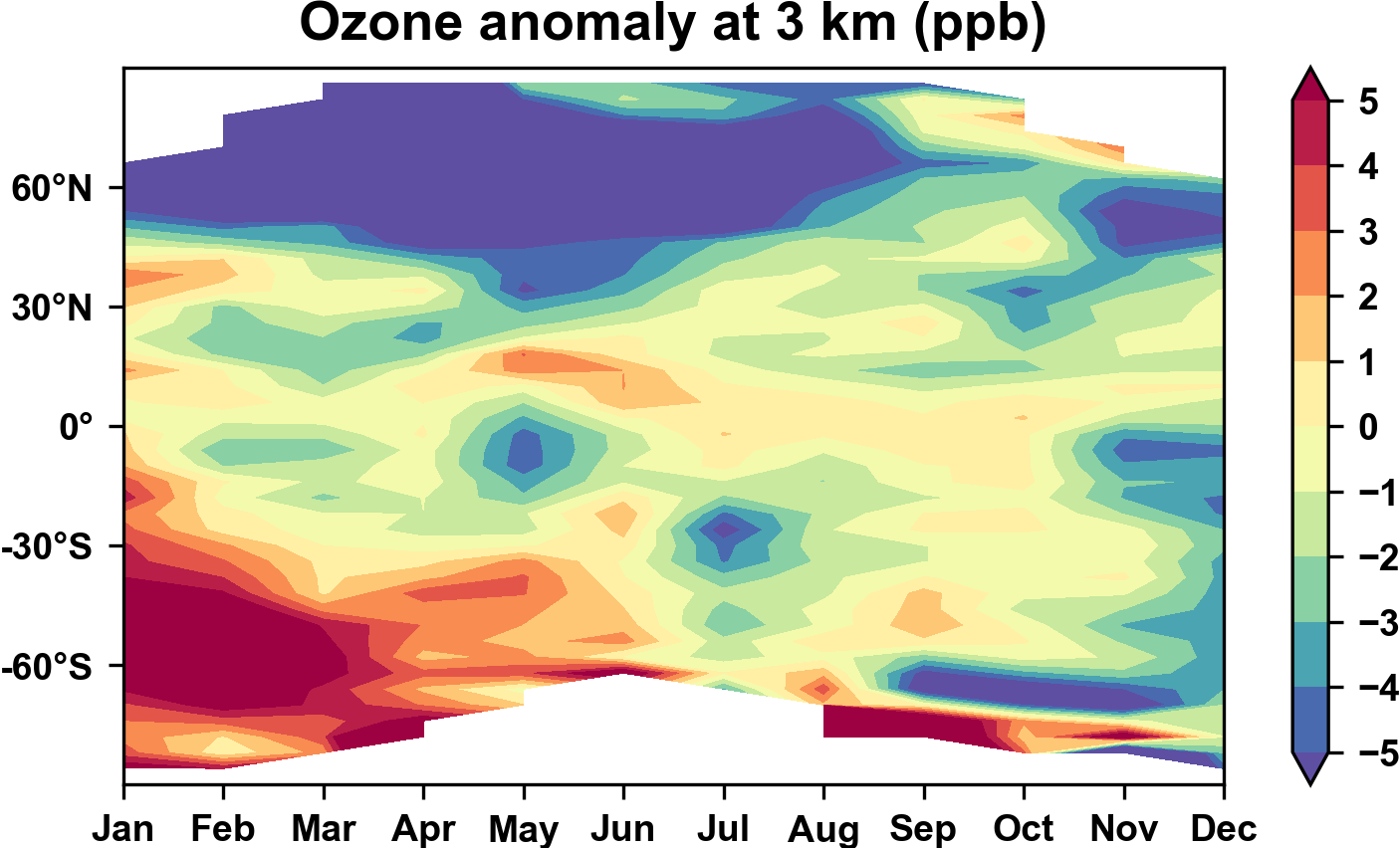


**Figure S6.** Monthly changes in ozone concentration at 3 km observed by IASI + GOME2 [53] between 2019 and 2020 as a function of latitude. Values are computed by subtracting 2019 values from 2020.


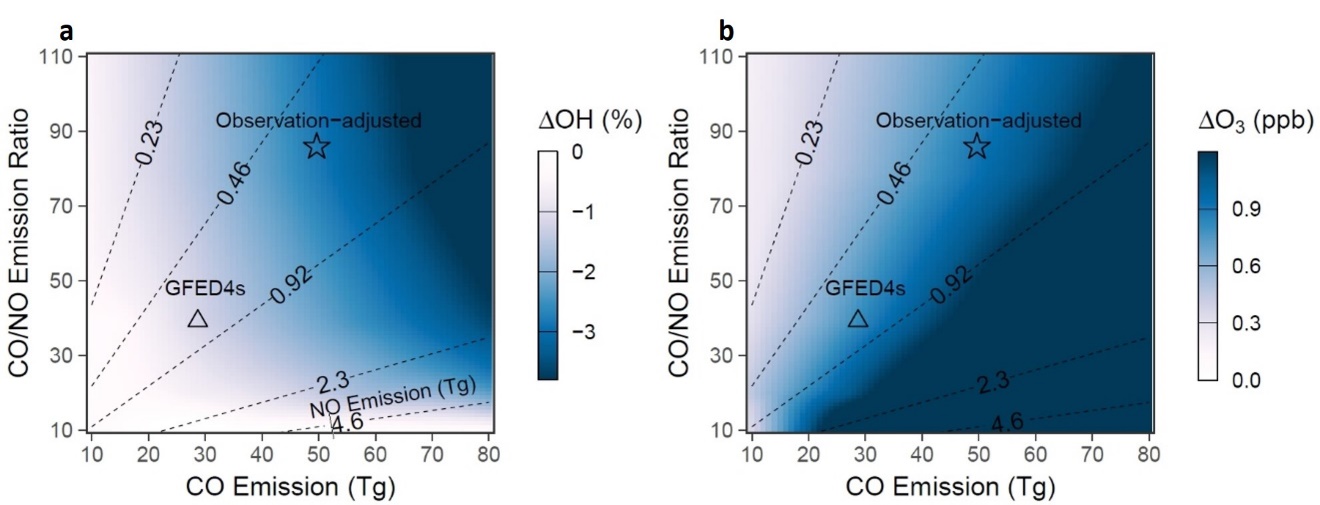


**Figure S7.** Southern hemispheric OH (a) and ozone (b) anomalies in January 2020 as a function of CO emissions and CO/NO emission ratios during the extreme Australian fire. The results are derived from a series of GEOS-Chem full-chemistry simulations by perturbing fire activity (emission magnitude) and fire combustion conditions (CO/NO ratio) relative to the base simulation. Tropospheric ozone anomalies are computed as hemispheric average at 3 km. Dashed lines represent NO emissions from the fire event. Triangle signs indicate Australian fire emissions from the GFED4s inventory [11], and star signs Australian fire emissions adjusted based on observational evidence [46, 47] (See Methods).


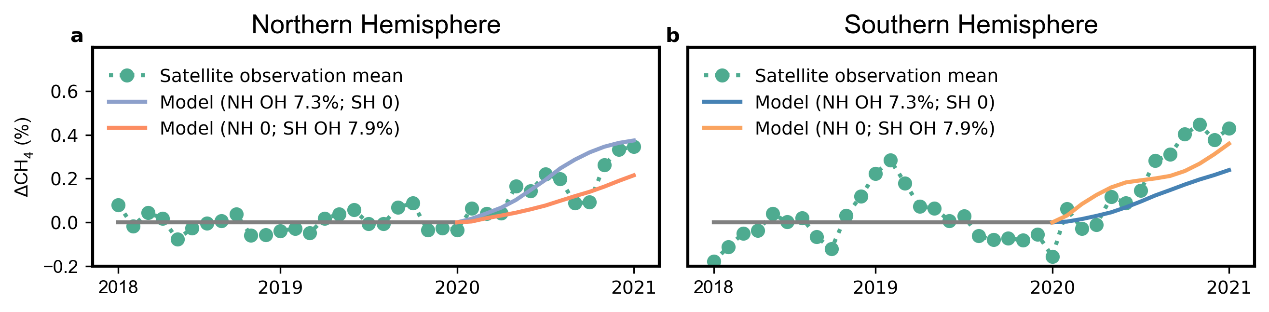


**Figure S8.** Impact of OH reduction on 2020 methane growth anomalies in the Northern (a) and Southern (b) Hemispheres. Anomalies observed from the GOSAT satellite instrument (green lines) are compared with those simulated by the GEOS-Chem CH_4_-only model under hypothetical scenarios that the global averaged 4.0% OH reduction occurs only in the Northern (blue lines) or Southern (orange lines) Hemispheres. The derivation of growth rate anomalies is illustrated in Figure S9.


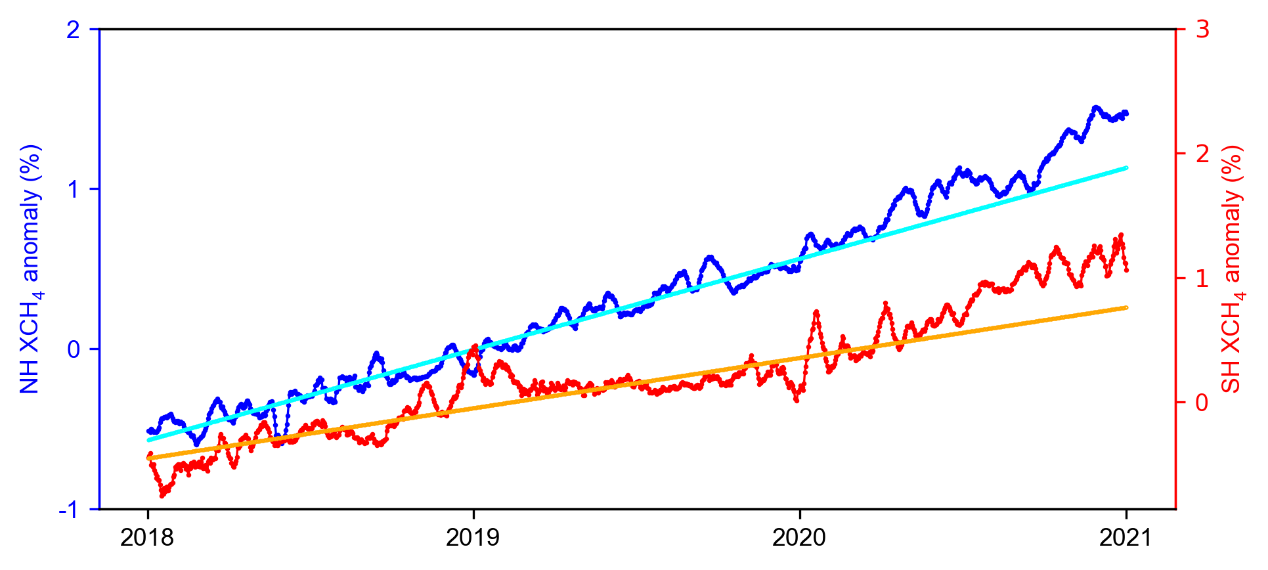


**Figure S9.** Derivation of methane growth rate anomalies shown in Figure 4 and Figure S8 from GOSAT satellite XCH_4_ observations. Blue (Northern Hemisphere) and red (Southern Hemisphere) lines show daily deseasonalized hemispheric methane anomalies relative to the 2018-2019 mean. Cyan (Northern Hemisphere) and orange (Southern Hemisphere) lines are linear fits to 2018-2019 data. Growth rate anomalies are computed by subtracting the 2018-2019 linear fits (cyan or orange lines) from the daily deseasonalized methane anomaly time series (blue or red lines).


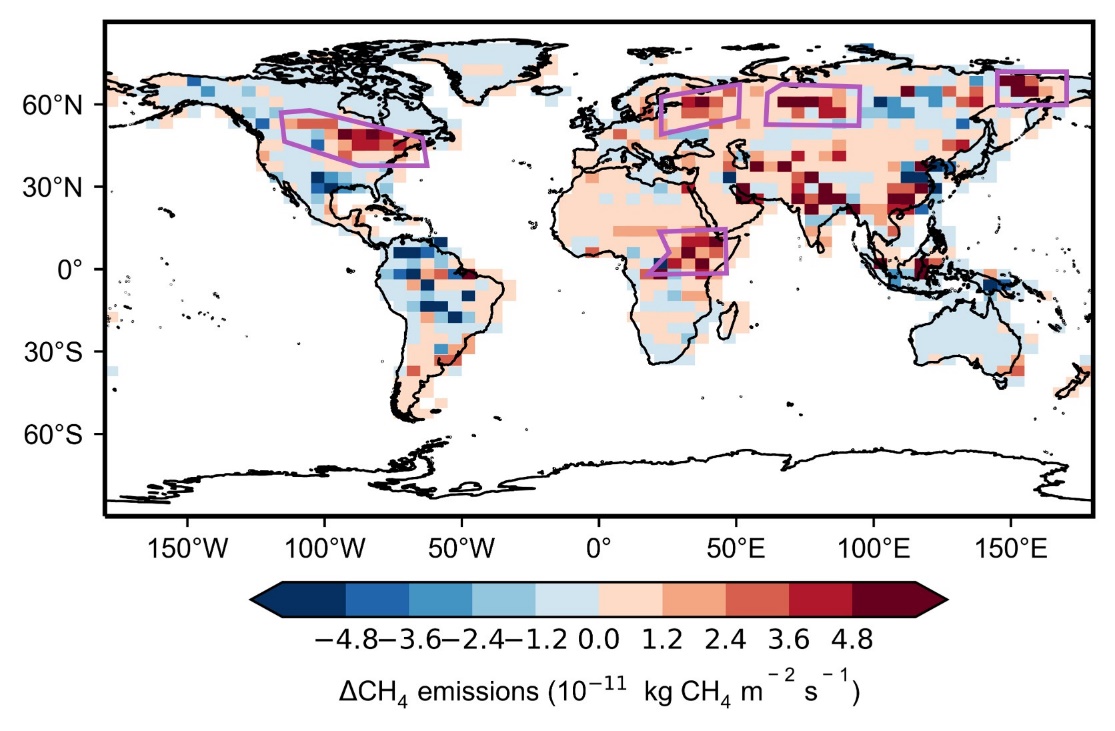


**Figure S10.** Changes of methane emission fluxes in 2020 relative to 2018-2019 inferred from an inversion of GOSAT methane column observations. Purple boxes denote increased methane emissions in regions with extensive wetlands.


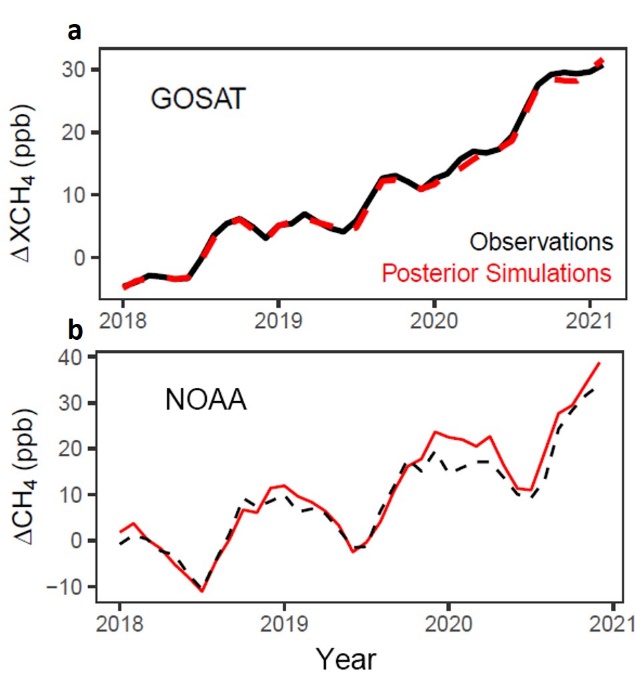


**Figure S11.** Comparison of methane growth rates between observations and posterior simulations. a, GOSAT observations used in the inverse analysis; b, NOAA observations used for independent evaluation.


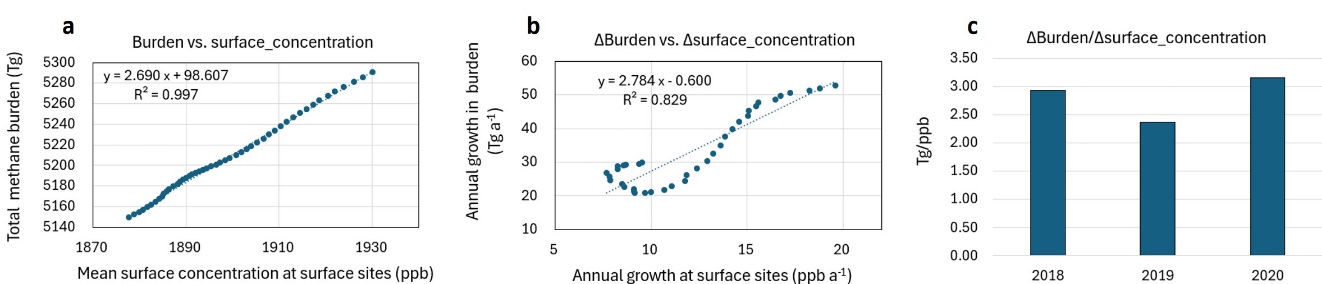


**Figure S12.** Conversion factors between annual changes in surface concentrations and those in the atmospheric burden derived from chemical transport model simulations. a, Mean surface concentrations vs. total methane burden. b, Annual growth in surface concentrations vs. annual growth in the atmospheric methane burden. c, Conversion factors for 2018, 2019, and 2020 from the posterior simulation of the inversion.


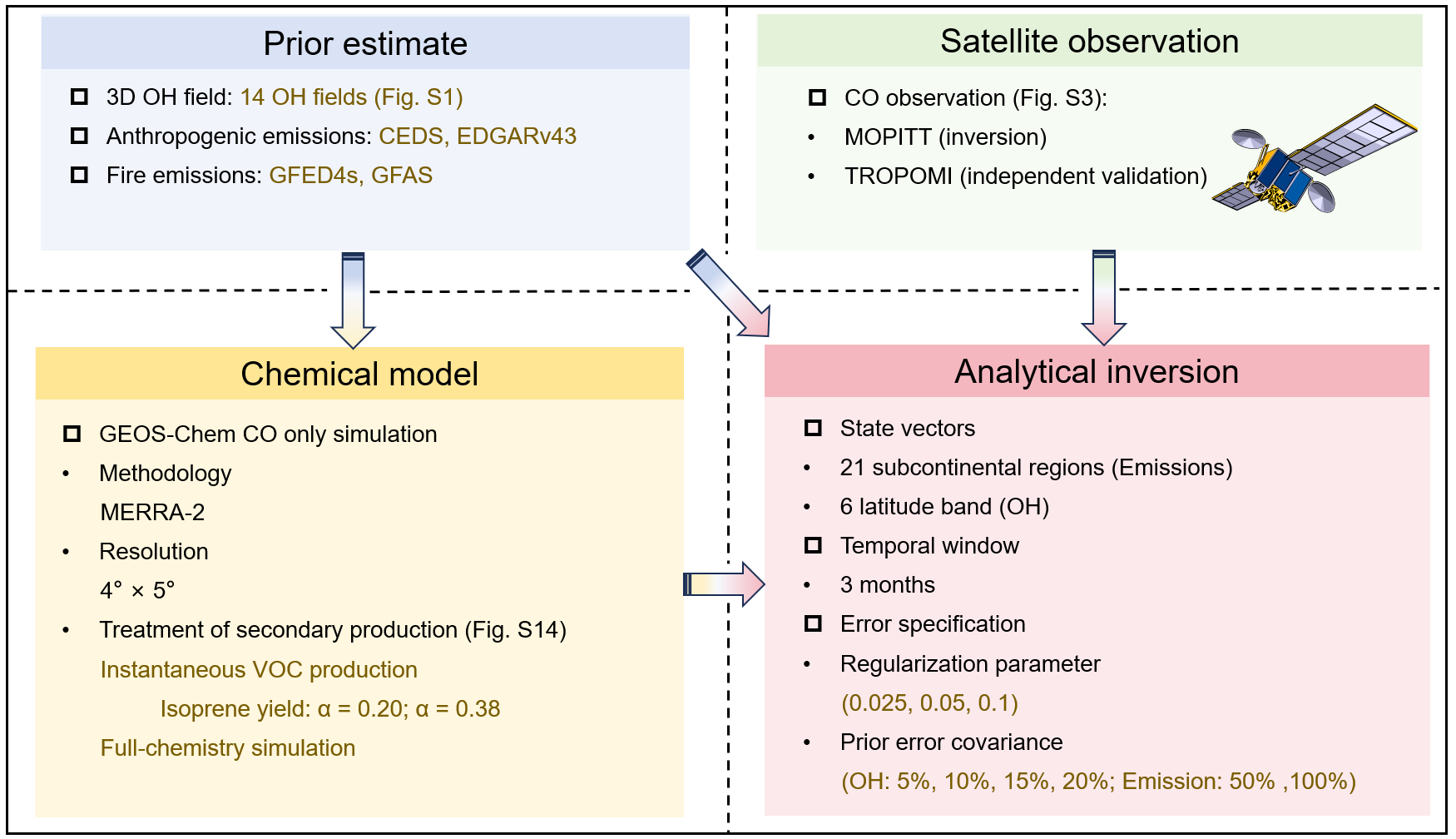


**Figure S13.** Methodological framework of the CO method. Brown texts denote sensitivity tests shown in Fig. S15a.


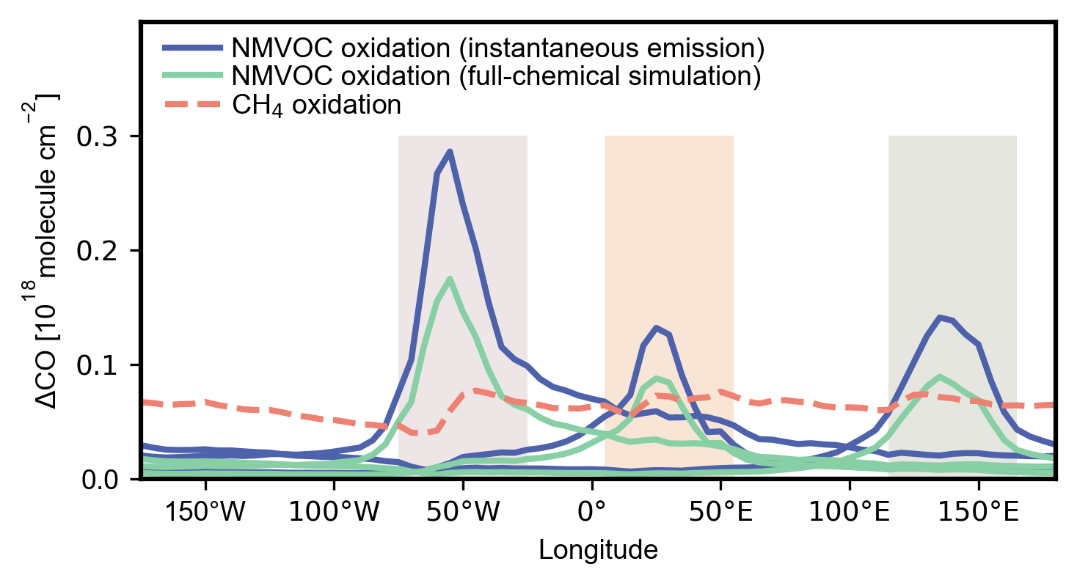


**Figure S14.** The sensitivities of CO column densities to oxidation of methane and NMVOC from South America, South Africa, and Oceania in the 0-30°S latitude band. The effect of parameterizing NMVOC oxidations as instantaneous emissions is evaluated against a full-chemistry simulation. Note the comparison is for the shape of the curves rather than the absolute magnitude. The shape of the curves is related to the signature of CO column concentrations due to NMVOC or CH_4_ oxidation. The average difference in the absolute magnitudes between instantaneous emission and full-chemistry simulation is due to differences in NMVOC emission inventories, and will be corrected in our algorithm through the optimization of general emissions ($\tilde{E}$).


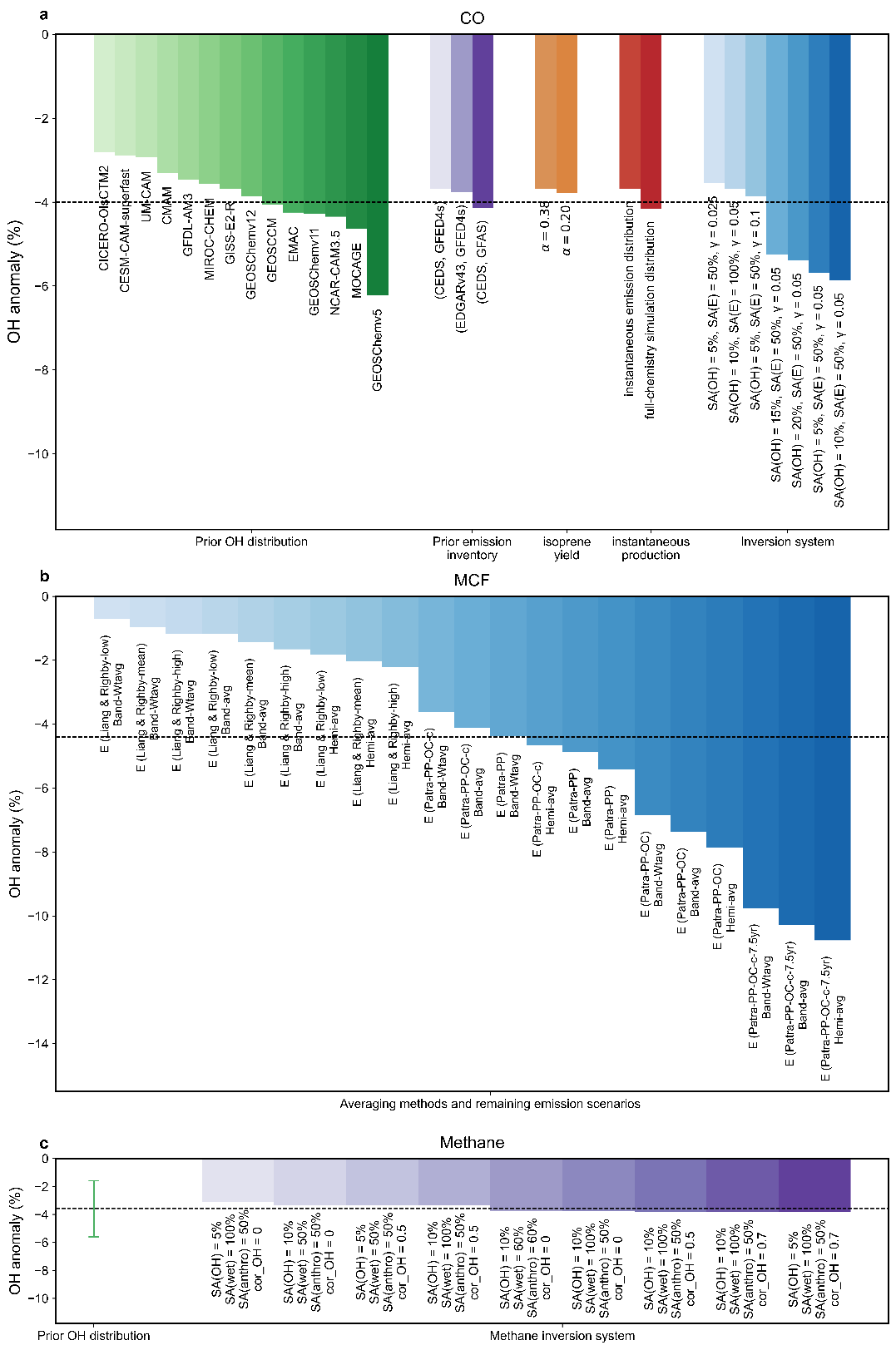


**Figure S15.** Inferred OH anomaly in 2020 across various sensitivity tests based on CO (a), MCF (b), and methane (c) methods. Error bar in Panel (c) represents errors of the methane method reported by Zhang et al. [33]. Labels corresponding to each bar denote the sensitivity parameters of the tests (See Methods). Gray dashed lines represent the ensemble mean.

_
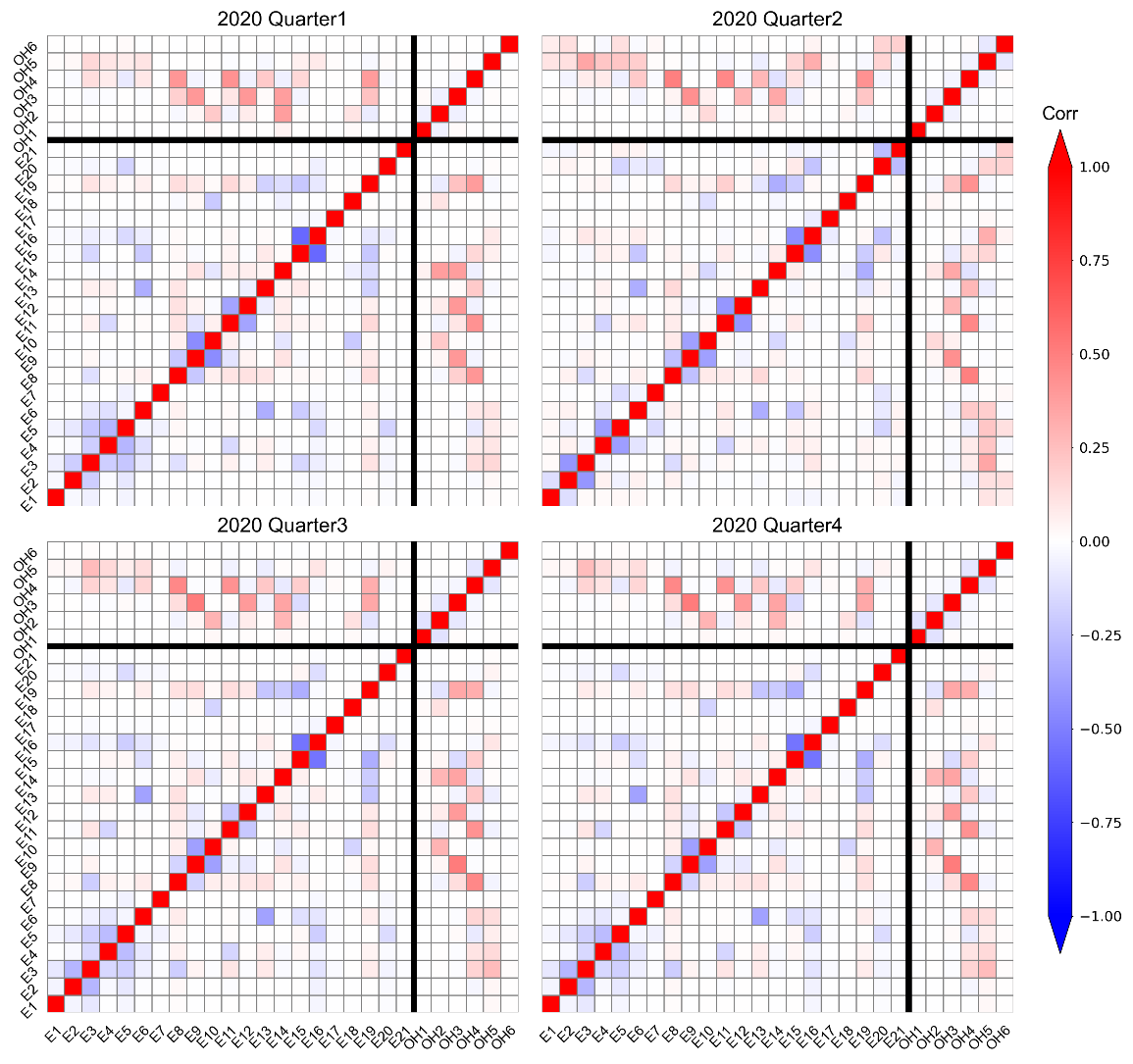
_

**Figure S16.** Posterior error correlations between generalized emissions from 21 sub-continental regions (Fig. S2) and OH in 6 latitudinal bands.


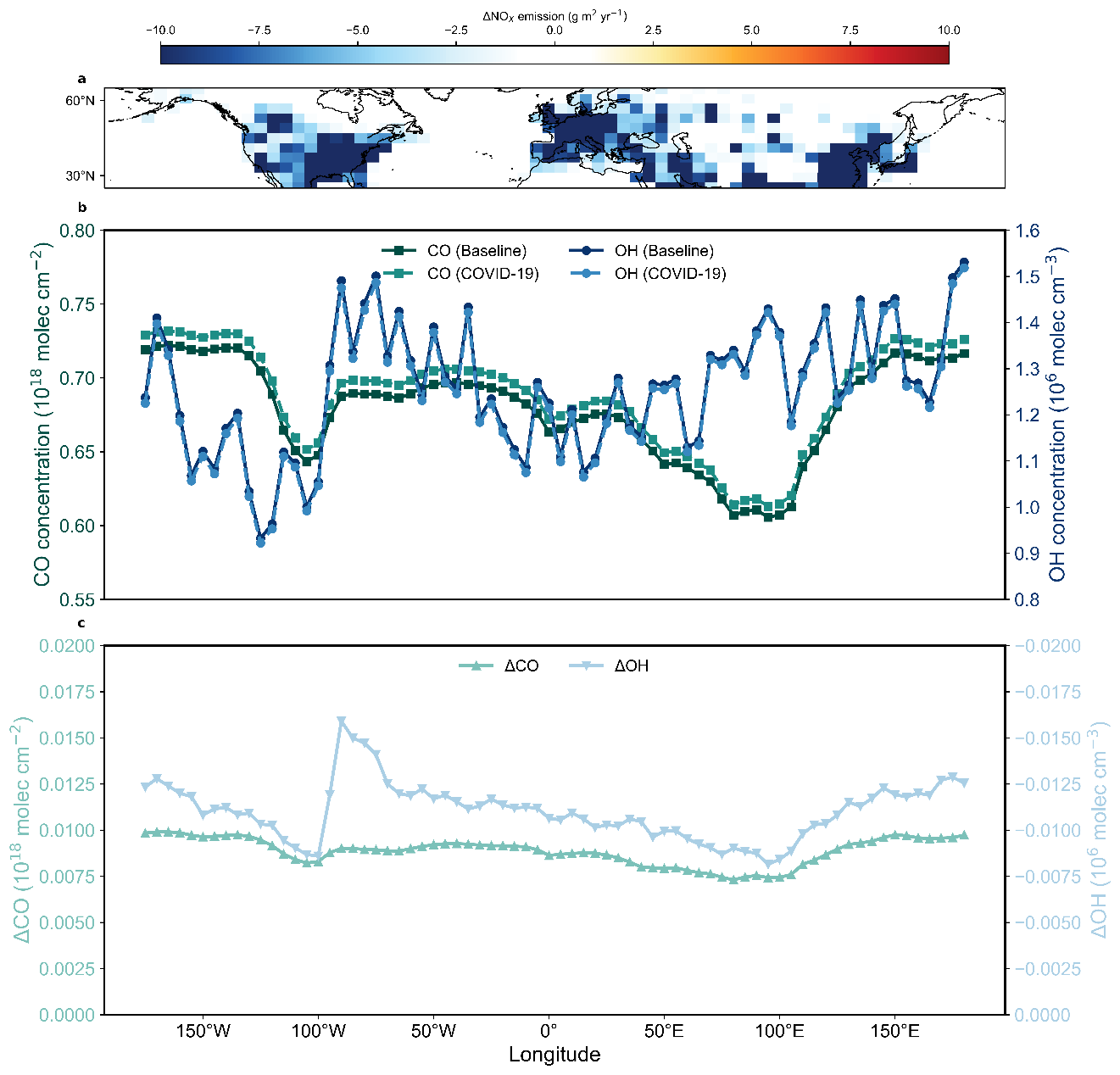


**Figure S17.** CO and OH sensitivity to COVID-19 lockdowns NOx reduction-only Scenario. a, COVID-19 NO_x_ emission reduction in May over the 30°N-60°N latitude band. b, CO and OH concentrations in May under both baseline and COVID-19 lockdowns NO_x_ emissions reductions scenarios within the 30°N-60°N latitude band in May. c, Differences in CO and OH concentrations between the baseline simulation and COVID-19 NO_x_ reduction emissions reductions scenario in May.


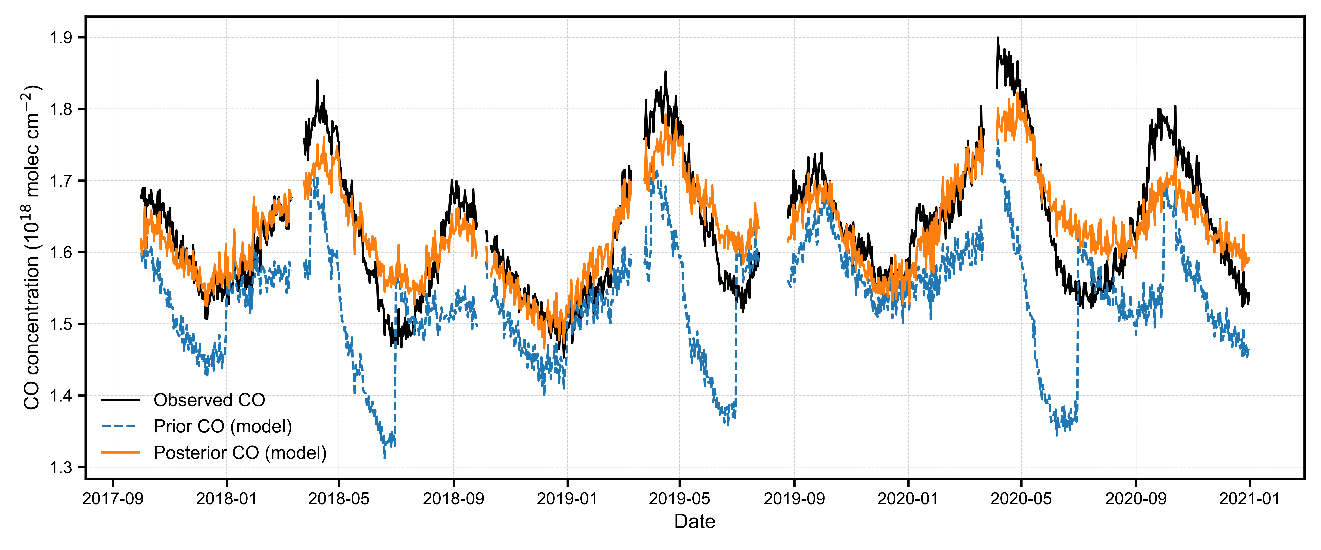


**Figure S18.** Daily time series of CO column concentrations from September 2017 to December 2020. Black line shows satellite-observed CO, the blue dashed line represents the prior model simulation, and the orange line indicates the posterior simulation after inverse modeling.


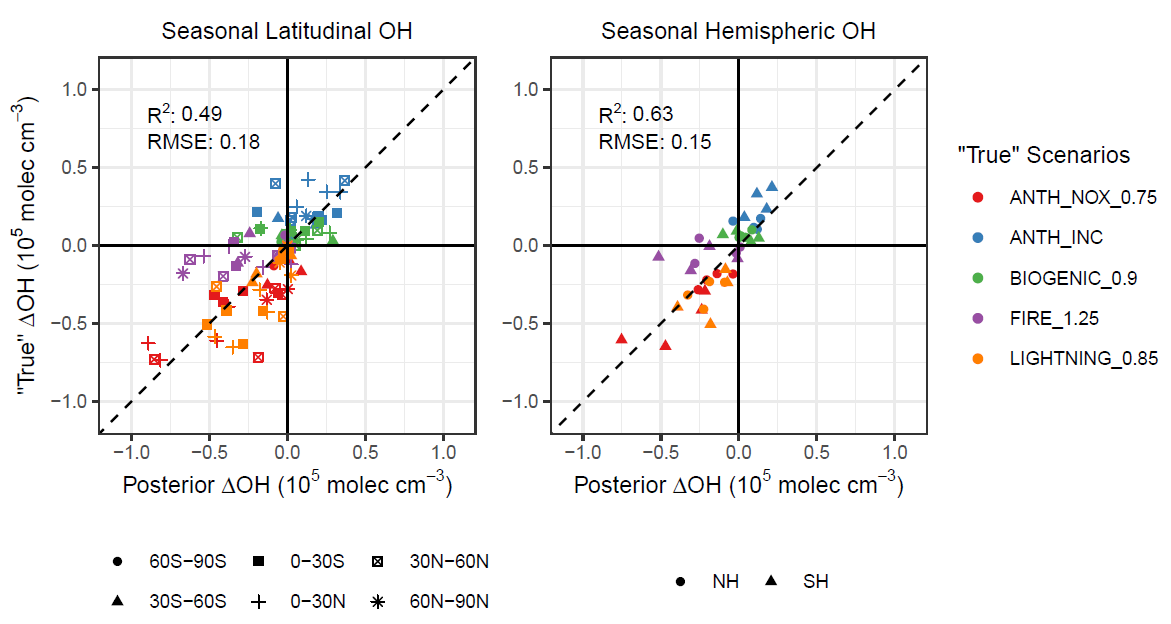


**Figure S19.** Ability of the proposed method to retrieve small seasonal changes in OH concentration assessed by OSSE. Results are shown for left 6 latitude bands and (right) two hemispheres. Colors represent various perturbation scenarios summarized in Table S4. Each symbol represents a seasonal result, and the OSSE is performed for four seasons.

**Table S1.** Varied configurations of GEOS-Chem simulations applied in the study.

| Simulation | Description |
| --- | --- |
| CO only simulation | Used in the inverse analysis of satellite CO observations to estimate continental-scale CO emissions and zonal OH concentrations.  The “CO-only” simulation solves the continuity equation for CO (Eq. 1) on a global 4^o^×5^o^ grid. The simulation uses a prescribed monthly 3-D OH concentration field from a full-chemistry simulation and a prescribed methane field constrained by global surface observations. The simulation computes the production of CO from the CH_4_+OH reaction and the removal of CO by OH oxidation.  The simulation accounts for primary CO emissions and secondary production from short-lived VOCs. The production from short-lived VOCs is treated as instantaneous CO emissions based on precursor emissions and their CO yields (see the “Forward model” section in Supplementary Text 1 and Table S2).  The simulation is driven by MERRA-2 reanalysis meteorology. |
| CH_4_ only simulation | Used in the simulation of global methane concentration distribution and inverse analysis of satellite methane observations to estimate the distribution of global methane emissions.  The “CH_4_-only” simulation solves the continuity equation for CH_4_ on a global 4^o^×5^o^ grid. The simulation uses a prescribed monthly 3-D OH concentration field from a full-chemistry simulation to compute the removal of CH_4_ by OH oxidation.  The simulation accounts for CH_4_ emissions from multiple natural and anthropogenic sources and CH_4_ removal by soil absorption.  The simulation is driven by MERRA-2 reanalysis meteorology. |
| Tropchem simulation | Used to understand the chemical response of OH and ozone to perturbations in anthropogenic and fire emissions.  The “tropchem” simulation is a full-chemistry simulation which solve the continuity equation jointly for a suite of gas and aerosol species on a global 4^o^×5^o^ grid and accounts for their natural and anthropogenic emissions, atmospheric transport, chemical transformation, and dry and wet deposition. The chemical mechanism involves ~ 200 species (including O_3_, HO_x_, NO_x_, CO, VOCs, and aerosol compositions) and ~ 800 reactions. The 3-D OH concentration field is simulated explicitly by computing dynamically its chemical sources and sinks. |

**Table S2.** Yields or scaling factors for reactive NMVOCs applied in the CO-only simulations.

| Source Type | Species | Yield^a^ |
| --- | --- | --- |
| Biogenic | Isoprene | 0.38^c^ |
|  | Acetone | 0.67^d^ |
|  | Monoterpenes | 0.2^d^ |
|  | Methanol | 1.0^d^ |
|  |  | Scaling Factor^b^ |
| Anthropogenic |  | 0.19^d^ |
| Biomass burning |  | 0.11^d^ |

^a^ Yields are applied to NMVOC emissions from the MEGAN model to compute CO formation from these biogenic species.

^b^ Scaling factors are applied to CO emissions from anthropogenic activities and biomass burning to compute CO formation from co-emitted reactive NMVOCs.

^c^ Value taken from Bates et al. [15].

^d^ Values taken from Fisher et al. [14].

**Table S3.** Global atmospheric CO sources derived from our inverse analyses^a^. Averages and ranges (in brackets) from the inversion ensemble are shown. Results are also grouped by source types (primary vs. secondary) and by atmospheric signature classes (enhanced signature near continents vs. uniform signature along a latitude band). Unit: Tg a^-1^.

| CO sources | Anthropogenic | Wildfire | NMVOC+OH | CH_4_+OH |
| --- | --- | --- | --- | --- |
|  | 505 [436-618] | 332 [274-387] | 651 [501-782] | 854 [763-981] |
| Group by primary or secondary | Primary Emissions | | Secondary Formation | |
|  | 837 [727-1005] | | 1505 [1332-1734] | |
| Group by atmospheric signature | Continent | | | Latitude-band |
|  | 1488 [1231-1787] | | | 854 [763-981] |

^a^ Values from our analysis are within the ranges reported in previous global CO studies summarized in Supplementary Table S4 of Zheng et al. [7].

**Table S4.** OSSE conditions.

|  | “True” atmosphere simulation^a^ | Inversion simulation |
| --- | --- | --- |
| Simulation mode | Tropchem simulation^b^ | CO-only simulation^b^ |
| Meteorology field^c^ | MERRA-2 | GEOS-FP |
| OH field | Online simulation | Prescribed field^d^ |
| Anthropogenic emissions^e^ | CEDS | EDGAR v4.3.2 |
| Fire emissions^e^ | GFED4s | GFAS |
| NMVOC production^e^ | Online simulation | Instantaneous production |

^a^ See Table S5 for the configurations of multiple “true” simulations.

^b^ See Table S1 for more information on different modes of GEOS-Chem.

^c^ Differences in meteorological data introduce systematic errors for the inversion. Figure 3 of Zhang et al. [33] shows differences on regional and hemispheric scales by comparing GEOS-Chem simulations driven by MERRA-2 and GEOS-FP.

^d^ Prescribed field is from a previous GEOS-Chem simulation, which differs substantially from the current version in terms of the chemical mechanism and emission inventories.

^e^ “True” simulations by the “tropchem” mode accounts for emissions of various species (e.g., NO_x_, CO, VOC, primary aerosols). Inversion simulations by the “CO-only” mode accounts for only emissions of CO and its VOC precursors.

**Table S5.** Baseline and perturbed “true” simulations to generate “true” CO and OH concentration fields.

| Label | Description |
| --- | --- |
| BASELINE | Default anthropogenic and natural emissions of the “tropchem” GEOS-Chem simulation |
| ANTH_NOX_0.75 | Anthropogenic NO_x_ emissions reduced by 25% |
| ANTH_INC | Anthropogenic NO_x_ emissions increased by 25% and anthropogenic CO and NMVOC emissions by 10% |
| BIOGENIC_0.9 | Biogenic isoprene emissions reduced by 10% |
| FIRE_1.25 | Biomass burning emissions, including NO_x_, CO and, NMVOC, increased by 25% |
| LIGHTNING_0.85 | Lightning NO_x_ emissions reduced by 15% |

# References

1. Deeter MN, Edwards DP, Francis GL *et al.* Radiance-based retrieval bias mitigation for the MOPITT instrument: the version 8 product. *Atmos Meas Tech* 2019; **12**(8): 4561-4580.

2. Jiang Z, Worden JR, Worden H *et al.* A 15-year record of CO emissions constrained by MOPITT CO observations. *Atmos Chem Phys* 2017; **17**(7): 4565-4583.

3. Deeter M, Francis G, Gille J *et al.* The MOPITT Version 9 CO product: sampling enhancements and validation. *Atmos Meas Tech* 2022; **15**(8): 2325-2344.

4. Hedelius JK, He T-L, Jones D *et al.* Evaluation of MOPITT Version 7 joint TIR–NIR X_CO_ retrievals with TCCON. *Atmos Meas Tech* 2019; **12**(10): 5547-5572.

5. Buchholz RR, Deeter MN, Worden HM *et al.* Validation of MOPITT carbon monoxide using ground-based Fourier transform infrared spectrometer data from NDACC. *Atmos Meas Tech* 2017; **10**(5): 1927-1956.

6. Tang W, Worden HM, Deeter MN *et al.* Assessing Measurements of Pollution in the Troposphere (MOPITT) carbon monoxide retrievals over urban versus non-urban regions. *Atmos Meas Tech* 2020; **13**(3): 1337-1356.

7. Zheng B, Chevallier F, Yin Y *et al.* Global atmospheric carbon monoxide budget 2000–2017 inferred from multi-species atmospheric inversions. *Earth Syst Sci Data* 2019; **11**(3): 1411-1436.

8. Hoesly RM, Smith SJ, Feng L *et al.* Historical (1750–2014) anthropogenic emissions of reactive gases and aerosols from the Community Emissions Data System (CEDS). *Geosci Model Dev* 2018; **11**(1): 369-408.

9. Li M, Zhang Q, Kurokawa J-i *et al.* MIX: a mosaic Asian anthropogenic emission inventory under the international collaboration framework of the MICS-Asia and HTAP. *Atmos Chem Phys* 2017; **17**(2): 935-963.

10. Marais EA, Wiedinmyer C. Air quality impact of diffuse and inefficient combustion emissions in Africa (DICE-Africa). *Environ Sci Technol* 2016; **50**(19): 10739-10745.

11. van der Werf GR, Randerson JT, Giglio L *et al.* Global fire emissions estimates during 1997–2016. *Earth Syst Sci Data* 2017; **9**(2): 697-720.

12. Guenther A, Jiang X, Heald CL *et al.* The Model of Emissions of Gases and Aerosols from Nature version 2.1 (MEGAN2.1): an extended and updated framework for modeling biogenic emissions. *Geosci Model Dev* 2012; **5**(6): 1471-1492.

13. Duncan BN, Logan JA, Bey I *et al.* Global budget of CO, 1988–1997: Source estimates and validation with a global model. *J Geophys Res* 2007; **112**(D22).

14. Fisher JA, Murray LT, Jones DBA *et al.* Improved method for linear carbon monoxide simulation and source attribution in atmospheric chemistry models illustrated using GEOS-Chem v9. *Geosci Model Dev* 2017; **10**(11): 4129-4144.

15. Bates KH, Jacob DJ. A new model mechanism for atmospheric oxidation of isoprene: global effects on oxidants, nitrogen oxides, organic products, and secondary organic aerosol. *Atmos Chem Phys* 2019; **19**(14): 9613-9640.

16. Naik V, Voulgarakis A, Fiore AM *et al.* Preindustrial to present-day changes in tropospheric hydroxyl radical and methane lifetime from the Atmospheric Chemistry and Climate Model Intercomparison Project (ACCMIP). *Atmos Chem Phys* 2013; **13**(10): 5277-5298.

17. Wecht KJ, Jacob DJ, Frankenberg C *et al.* Mapping of North American methane emissions with high spatial resolution by inversion of SCIAMACHY satellite data. *J Geophys Res: Atmos* 2014; **119**(12): 7741-7756.

18. Gelaro R, McCarty W, Suárez MJ *et al.* The modern-era retrospective analysis for research and applications, version 2 (MERRA-2). *J Clim* 2017; **30**(14): 5419-5454.

19. Murray LT. Lightning NO_X_ and impacts on air quality. *Current Pollution Reports* 2016; **2**(2): 115-133.

20. Lan X, E. J. Dlugokencky, J. W. Mund, A. W. Crotwell, M. J, Crotwell, E. Moglia, M. Madronich, D. Neff, and K.W. Thoning (2022). Atmospheric Methane Dry Air Mole Fractions from the NOAA GML Carbon Cycle Cooperative Global Air Sampling Network, 1983-2021, Version: 2022-11-21, https://doi.org/10.15138/VNCZ-M766.

21. Brasseur GP, Jacob DJ. *Modeling of atmospheric chemistry*: Cambridge University Press, 2017.

22. Hansen PC. The L-curve and its use in the numerical treatment of inverse problems. 1999.

23. Lamboll RD, Jones CD, Skeie RB *et al.* Modifying emissions scenario projections to account for the effects of COVID-19: protocol for CovidMIP. *Geosci Model Dev* 2021; **14**(6): 3683-3695.

24. Doumbia T, Granier C, Elguindi N *et al.* Changes in global air pollutant emissions during the COVID-19 pandemic: a dataset for atmospheric modeling. *Earth Syst Sci Data* 2021; **13**(8): 4191-4206.

25. Montzka SA, Spivakovsky CM, Butler JH *et al.* New observational constraints for atmospheric hydroxyl on global and hemispheric scales. *Science* 2000; **288**(5465): 500-503.

26. Montzka SA, Krol M, Dlugokencky E *et al.* Small interannual variability of global atmospheric hydroxyl. *Science* 2011; **331**(6013): 67-69.

27. Patra P, Krol M, Prinn R *et al.* Methyl chloroform continues to constrain the hydroxyl (OH) variability in the troposphere. *J Geophys Res: Atmos* 2021; **126**(4): e2020JD033862.

28. Liang Q, Rigby M, Fang X *et al.* Hydrofluorocarbons (HFCs). *SCIENTIFIC ASSESSMENT OF OZONE DEPLETION 2022*: World Meteorological Organization; 2022. 119-151.

29. Penn E, Jacob DJ, Chen Z *et al.* What can we learn about tropospheric OH from satellite observations of methane? *Atmos Chem Phys* 2025; **25**(5): 2947-2965.

30. Parker RJ, Webb A, Boesch H *et al.* A decade of GOSAT Proxy satellite CH_4_ observations. *Earth Syst Sci Data* 2020; **12**(4): 3383-3412.

31. Zhang Y, Jacob DJ, Lu X *et al.* Attribution of the accelerating increase in atmospheric methane during 2010–2018 by inverse analysis of GOSAT observations. *Atmos Chem Phys* 2021; **21**(5): 3643-3666.

32. Maasakkers JD, Jacob DJ, Sulprizio MP *et al.* Global distribution of methane emissions, emission trends, and OH concentrations and trends inferred from an inversion of GOSAT satellite data for 2010–2015. *Atmos Chem Phys* 2019; **19**(11): 7859-7881.

33. Zhang Y, Jacob DJ, Maasakkers JD *et al.* Monitoring global tropospheric OH concentrations using satellite observations of atmospheric methane. *Atmos Chem Phys* 2018; **18**(21): 15959-15973.

34. Qu Z, Jacob DJ, Zhang Y *et al.* Attribution of the 2020 surge in atmospheric methane by inverse analysis of GOSAT observations. *Environ Res Lett* 2022; **17**(9): 094003.

35. Peng S, Lin X, Thompson RL *et al.* Wetland emission and atmospheric sink changes explain methane growth in 2020. *Nature* 2022; **612**(7940): 477-482.

36. Feng L, Palmer PI, Parker RJ *et al.* Methane emissions are predominantly responsible for record-breaking atmospheric methane growth rates in 2020 and 2021. *Atmos Chem Phys* 2023; **23**(8): 4863-4880.

37. Zhang Z, Poulter B, Feldman AF *et al.* Recent intensification of wetland methane feedback. *Nat Clim Change* 2023; **13**(5): 430-433.

38. Laughner JL, Cohen RC. Direct observation of changing NO_x_ lifetime in North American cities. *Science* 2019; **366**(6466): 723-727.

39. Zhu Q, Laughner JL, Cohen RC. Estimate of OH trends over one decade in North American cities. *Proc Natl Acad Sci USA* 2022; **119**(16): e2117399119.

40. Valin L, Russell A, Cohen RC. Variations of OH radical in an urban plume inferred from NO_2_ column measurements. *Geophys Res Lett* 2013; **40**(9): 1856-1860.

41. Liu F, Beirle S, Zhang Q *et al.* NO_x_ lifetimes and emissions of cities and power plants in polluted background estimated by satellite observations. *Atmos Chem Phys* 2016; **16**(8): 5283-5298.

42. Cooper MJ, Martin RV, Hammer MS *et al.* Global fine-scale changes in ambient NO_2_ during COVID-19 lockdowns. *Nature* 2022; **601**(7893): 380-387.

43. Ziemke JR, Kramarova NA, Frith SM *et al.* NASA Satellite Measurements Show Global-Scale Reductions in Free Tropospheric Ozone in 2020 and Again in 2021 During COVID-19. *Geophys Res Lett* 2022; **49**(15): e2022GL098712.

44. Miyazaki K, Bowman K, Sekiya T *et al.* Global tropospheric ozone responses to reduced NO_x_ emissions linked to the COVID-19 worldwide lockdowns. *Sci Adv* 2021; **7**(24): eabf7460.

45. Lelieveld J, Gromov S, Pozzer A *et al.* Global tropospheric hydroxyl distribution, budget and reactivity. *Atmos Chem Phys* 2016; **16**(19): 12477-12493.

46. van der Velde IR, van der Werf GR, Houweling S *et al.* Vast CO_2_ release from Australian fires in 2019-2020 constrained by satellite. *Nature* 2021; **597**(7876): 366-369.

47. Wan N, Xiong X, Kluitenberg GJ *et al.* Estimation of biomass burning emission of NO_2_ and CO from 2019–2020 Australia fires based on satellite observations. *Atmos Chem Phys* 2023; **23**(1): 711-724.

48. Saunois M, Martinez A, Poulter B *et al.* Global methane budget 2000–2020. *Earth Syst Sci Data* 2025; **17**(5): 1873-1958.

49. Prather MJ, Holmes CD, Hsu J. Reactive greenhouse gas scenarios: Systematic exploration of uncertainties and the role of atmospheric chemistry. *Geophys Res Lett* 2012; **39**(9).

50. Laughner JL, Neu JL, Schimel D *et al.* Societal shifts due to COVID-19 reveal large-scale complexities and feedbacks between atmospheric chemistry and climate change. *Proc Natl Acad Sci USA* 2021; **118**(46): e2109481118.

51. Stevenson DS, Derwent RG, Wild O *et al.* COVID-19 lockdown emission reductions have the potential to explain over half of the coincident increase in global atmospheric methane. *Atmos Chem Phys* 2022; **22**(21): 14243-14252.

52. Skeie RB, Hodnebrog Ø, Myhre G. Trends in atmospheric methane concentrations since 1990 were driven and modified by anthropogenic emissions. *Commun Earth Environ* 2023; **4**(1): 317.

53. Cuesta J, Eremenko M, Liu X *et al.* Satellite observation of lowermost tropospheric ozone by multispectral synergism of IASI thermal infrared and GOME-2 ultraviolet measurements over Europe. *Atmos Chem Phys* 2013; **13**(19): 9675-9693.
